# Essential role of pyrophosphate homeostasis mediated by the pyrophosphate-dependent phosphofructokinase in *Toxoplasma gondii*

**DOI:** 10.1371/journal.ppat.1010293

**Published:** 2022-02-01

**Authors:** Xuke Yang, Xiaoyan Yin, Jiaojiao Liu, Zhipeng Niu, Jichao Yang, Bang Shen

**Affiliations:** 1 State Key Laboratory of Agricultural Microbiology, College of Veterinary Medicine, Huazhong Agricultural University, Wuhan, China; 2 Key Laboratory of Preventive Medicine in Hubei Province, Huazhong Agricultural University, Wuhan, China; 3 Hubei Hongshan Laboratory, Wuhan, China; University of Georgia Athens, UNITED STATES

## Abstract

Many biosynthetic pathways produce pyrophosphate (PPi) as a by-product, which is cytotoxic if accumulated at high levels. Pyrophosphatases play pivotal roles in PPi detoxification by converting PPi to inorganic phosphate. A number of apicomplexan parasites, including *Toxoplasma gondii* and *Cryptosporidium parvum*, express a PPi-dependent phosphofructokinase (PPi-PFK) that consumes PPi to power the phosphorylation of fructose-6-phosphate. However, the physiological roles of PPi-PFKs in these organisms are not known. Here, we report that *Toxoplasma* expresses both ATP- and PPi-dependent phosphofructokinases in the cytoplasm. Nonetheless, only PPi-PFK was indispensable for parasite growth, whereas the deletion of ATP-PFK did not affect parasite proliferation or virulence. The conditional depletion of PPi-PFK completely arrested parasite growth, but it did not affect the ATP level and only modestly reduced the flux of central carbon metabolism. However, PPi-PFK depletion caused a significant increase in cellular PPi and decreased the rates of nascent protein synthesis. The expression of a cytosolic pyrophosphatase in the PPi-PFK depletion mutant reduced its PPi level and increased the protein synthesis rate, therefore partially rescuing its growth. These results suggest that PPi-PFK has a major role in maintaining pyrophosphate homeostasis in *T*. *gondii*. This role may allow PPi-PFK to fine-tune the balance of catabolism and anabolism and maximize the utilization efficiency for carbon nutrients derived from host cells, increasing the success of parasitism. Moreover, PPi-PFK is essential for parasite propagation and virulence *in vivo* but it is not present in human hosts, making it a potential drug target to combat toxoplasmosis.

## Author summary

Different from classic ATP-dependent phosphofructokinases, PPi-PFKs use pyrophosphate consumption to power the conversion of fructose-6-phosphate to fructose-1,6-bisphosphate, the committed step of glycolysis. PPi-PFK is found in diverse organisms including archaea, bacteria, protists and plants. However, half a century after its first discovery, the physiological functions of PPi-PFK are still not well defined. Using the *Toxoplasma gondii* parasite as a model, here we show that PPi-PFK has a coordinator function to assure matched activities of anabolism and catabolism. This is achieved by maintaining the homeostasis of PPi, which is a byproduct, as well as an inhibitor of many biosynthetic reactions. PPi-PFK hydrolyzes PPi to promote anabolism, meanwhile being a glycolytic enzyme involved in catabolism. As such, it gauges the anabolic and catabolic activities in parasites to maximize the utilization efficiency of acquired nutrients. This work provides important insights to understand the physiological significance of PPi-PFK in *Toxoplasma* and other organisms.

## Introduction

Inorganic pyrophosphate (PPi) is a ubiquitous metabolite in cells [1,2]. It can be generated by diverse biosynthetic reactions, including the synthesis of proteins, DNA, RNA, isoprenoids, polysaccharides, etc. [3–5]. PPi is largely treated as a by-product of anabolism, and its physiological roles are not fully defined. The abnormal accumulation of PPi is toxic to cells, likely because high levels of PPi are unfavorable for the reactions that generate this compound [6]. As such, cellular PPi levels are tightly regulated. One common mechanism for regulating PPi levels is through hydrolysis catalyzed by pyrophosphatases (PPases). There are two types of PPases, soluble PPases that convert PPi to inorganic phosphate (Pi) and membrane-bound H^+^/ Na^+^-pumping PPases that couple the hydrolysis of PPi to power the proton and/or Na^+^ transport across membranes [7–9]. In addition, in plants and some bacteria and protozoa, PPi hydrolysis can also be catalyzed by pyrophosphate-dependent phosphofructokinases (PPi-PFKs), which use PPi as the energy and phosphate donor to catalyze the conversion of fructose-6-phosphate (F6P) to fructose-1,6-bisphosphate (FBP) [10–13].

Most cells and organisms use ATP-dependent phosphofructokinases (ATP-PFKs) to catalyze the irreversible conversion of F6P to FBP [14]. By contrast, the reaction catalyzed by PPi-PFKs is reversible and can occur in both directions, suggesting that PPi-PFKs may function in both glycolysis and gluconeogenesis. PPi-PFK was first discovered in *Entamoeba histolytica*, a parasitic amoeba that infects humans [15]. Subsequently, it was found in diverse organisms, including archaea, bacteria, photosynthetic protists, higher plants and parasitic protozoa such as *Giardia*, *Trichomonas* and *Toxoplasma* [12,16–21]. Although the enzymatic activity and kinetics of a number of PPi-PFKs have been well studied, the physiological roles of PPi-PFKs are much less clear. Rice mutants with reduced PPi-PFK activity produced floury-like grain endosperm with reduced kernel thickness and total starch content without affecting the vegetative and reproductive growth of the plants [22]. Similarly, in other plants, such as sugarcane and tobacco, manipulating the level or activity of PPi-PFKs leads to altered metabolite levels but does not have a significant impact on the overall growth of plants [23,24]. In the methanotrophic bacterium *Methylotuvimicrobium alcaliphilum*, deletion of PPi-PFK reduced bacterial growth on methane or methanol, suggesting a role of PPi-PFK in C1 assimilation [25]. By contrast, the PPi-PFK in the methylotrophic actinomycete *Amycolatopsis methanolica* is expressed when cells are grown on glucose and completely replaced by ATP-PFK when grown on one-carbon compounds such as methanol [26]. The diverse functional modes of PPi-PFKs in different organisms may occur because they can work in either glycolysis or gluconeogenesis, and because different organisms have different combinations of ATP-PFKs, PPi-PFKs, PPases and fructose-1,6-bisphosphatases (FBPases) that are involved in F6P/FBP interconversion and PPi homeostasis.

*Toxoplasma gondii* is an obligate intracellular parasite infecting one-third of the world’s human population and numerous animals, and it has a complex carbon metabolism network that helps it to parasitize diverse host environments [27,28]. It has an intact glycolysis pathway. In addition, many glycolytic enzymes in *Toxoplasma* have two or more isoforms, each of which has a distinct expression pattern during the parasite’s life cycle [29,30]. Disrupting the primary glucose transporters that import host glucose into parasites or the first glycolytic enzyme hexokinase only has minor effects on the growth of tachyzoites (the parasite form during acute infection) [31,32]. This characteristic is explained by the ability of *Toxoplasma* to use glutamine as an alternative carbon source [33]. The depletion of cytosolic pyruvate kinase 1, which is required for the efficient use of both glucose and glutamine, arrests parasite growth [34]. Notably, *Toxoplasma* tachyzoites constitutively express two isoforms of the gluconeogenic enzyme FBPase in the cytosol, and FBP2 is essential for parasite growth even in the presence of glucose. It was proposed that futile cycling between F6P and FBP is a key regulatory mechanism in *Toxoplasma* for rapid adaptation to changing environments [35]. One unusual aspect of *Toxoplasma* metabolism is that its cellular PPi level is higher than that of ATP [36]. *T*. *gondii* expresses a cytosolic PPase that can convert PPi to Pi. The overexpression of this PPase results in a reduced concentration of cytosolic PPi. Unexpectedly, PPase overexpression led to higher glycolytic flux and increased cellular ATP levels but decreased the growth rate of the parasites [37]. The underlying mechanisms are not yet fully understood, but these results highlight a critical role of PPi in metabolic regulation in *T*. *gondii*.

The *Toxoplasma* genome encodes two phosphofructokinases. However, the vast majority of phosphofructokinase activity in tachyzoites was PPi-dependent, and the ATP-PFK activity was minimal when the crude extract was assayed [17]. PPi-PFK was also purified from *Toxoplasma*, and the enzymatic properties were estimated. Its activity is Mg^2+^-dependent and is not regulated by fructose 2,6-biphosphate [38]. Except for the biochemical characterization of these PFKs, little is known about their functions. In this study, we found that PPi-PFK is essential for *Toxoplasma* parasite growth both *in vitro* and *in vivo*, whereas ATP-PFK is dispensable. Further work suggested that PPi-PFK works together with the cytosolic PPase to regulate the PPi level, which helps to maintain a balance between catabolism and anabolism.

## Results

### *Toxoplasma* encodes two active PFKs that are ATP- or PPi-dependent respectively

**Fig 1:**
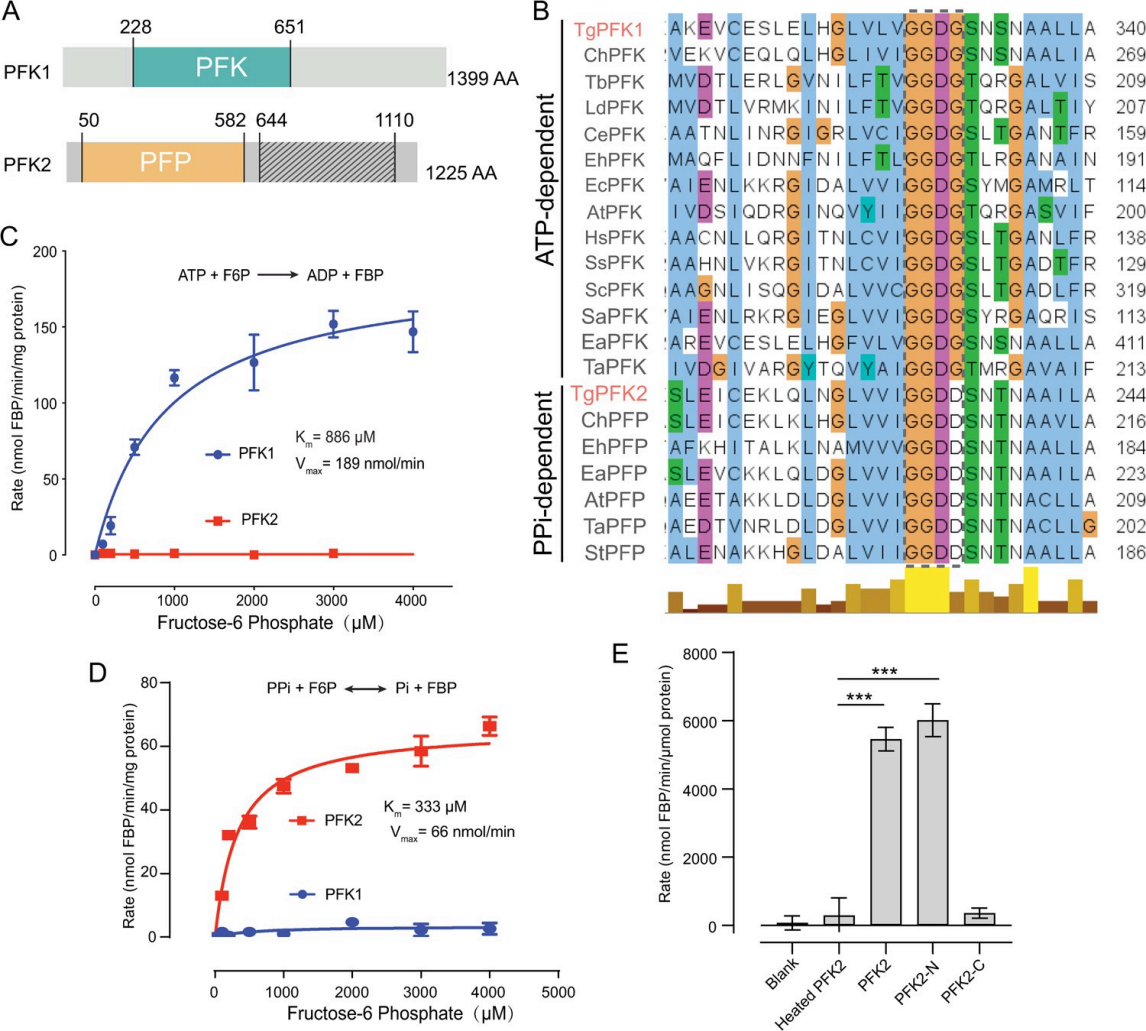
*Toxoplasma* expresses two PFKs that are PPi- and ATP-dependent. **A**, Conserved domains in *Toxoplasma* PFK1 and PFK2, as determined by BLAST analyses. PFK: phosphofructokinase, PFP: PPi-dependent phosphofructokinase. The shaded region (644–1110) in PFK2 is a pseudo-PFK domain that lacks key residues for activity. **B**, Sequence alignments of two types of PFKs from selected species. The “GGDD/G” motif that specifies the substrates is boxed in gray. **C-D**: ATP- and PPi-dependent activities of recombinant PFK1 and PFK2 when assayed under different F6P concentrations. Each assay was repeated three times independently. **E**, Enzymatic activity of the N (PFK2-N, 1–595) and C (PFK2-C, 597–1225) terminal domains of PFK2 in the presence of 4 mM F6P. PBS (Blank) and heat inactivated PFK2 (Heated PFK2) were included as negative controls. ***P < 0.001, paired student’s t-test (n = 3).

Two PFKs have been previously reported in *Toxoplasma*, namely PFK1 (TGGT1_240890) and PFK2 (TGGT1_226960) [29]. The existing transcriptomic data indicate that PFK1 expression is low in tachyzoites and is slightly increased in bradyzoites (ToxoDB.org). PFK2 is more actively transcribed than PFK1 in tachyzoites. Motif structure analyses showed that PFK1 contained a classic phosphofructokinase domain in the N-terminal part (Fig 1A). PFK2, on the other hand, has two putative phosphofructokinase domains. However, the one in the C-terminal half (amino acids 644–1110) apparently lacks the key residues for activity (Fig 1A). The one in the N-terminus is predicted to be an active phosphofructokinase. A closer look at the sequence signatures suggests that PFK2 is likely a PPi-PFK, because it has a “GGDD” motif in the active center that is commonly found in PPi-PFKs [39–41]. A “GGDG” motif in the same region correlated with ATP-PFKs (Fig 1B). Structural analyses indicate that the “GGDD/G” motif is at the ATP/PPi binding pocket of PFKs. As such, a smaller glycine residue would allow for the docking of the bulkier ATP, whereas the larger aspartic acid would make a smaller pocket that better fits PPi (S1 Fig). To examine the enzymatic activity of the two PFK proteins, the proteins were recombinantly expressed in *E*. *coli* and purified to homogeneity. Subsequent activity tests showed that PFK1 was active only when ATP was present, and the Km was 886 μM for F6P (Fig 1C). In contrast, PFK2 could only use PPi as the phosphoryl donor, and its Km for F6P was 333 μM (Fig 1D), close to what was reported previously [38]. The two putative phosphofructokinase domains of PFK2 were individually purified and assayed for the PPi dependent PFK activity. The results showed that the activity of the N-terminal domain (PFK2-N, 1–595) was similar to that of the full-length protein, whereas the C-terminal domain (597–1225) lacked detectable activity (Fig 1E). These results are consistent with the sequence analysis data (Fig 1A). Taken together, due to the sequence signatures and enzymatic activities, it was concluded that PFK1 in *Toxoplasma* is an ATP-PFK, whereas PFK2 is a PPi-PFK.

To examine the expression and localization of the two PFKs in *T*. *gondii*, an epitope tag (smHA for PFK1 and Ty for PFK2) was fused to the C-terminus of each protein at the endogenous locus. Immunofluorescence assays of the corresponding transgenic strains suggested that both PFK1 and PFK2 were localized to the parasite cytoplasm, as evidenced by colocalization with the cytoplasmic marker ALD (S2 Fig).

### The ATP-dependent PFK is dispensable for parasite growth

**Fig 2:**
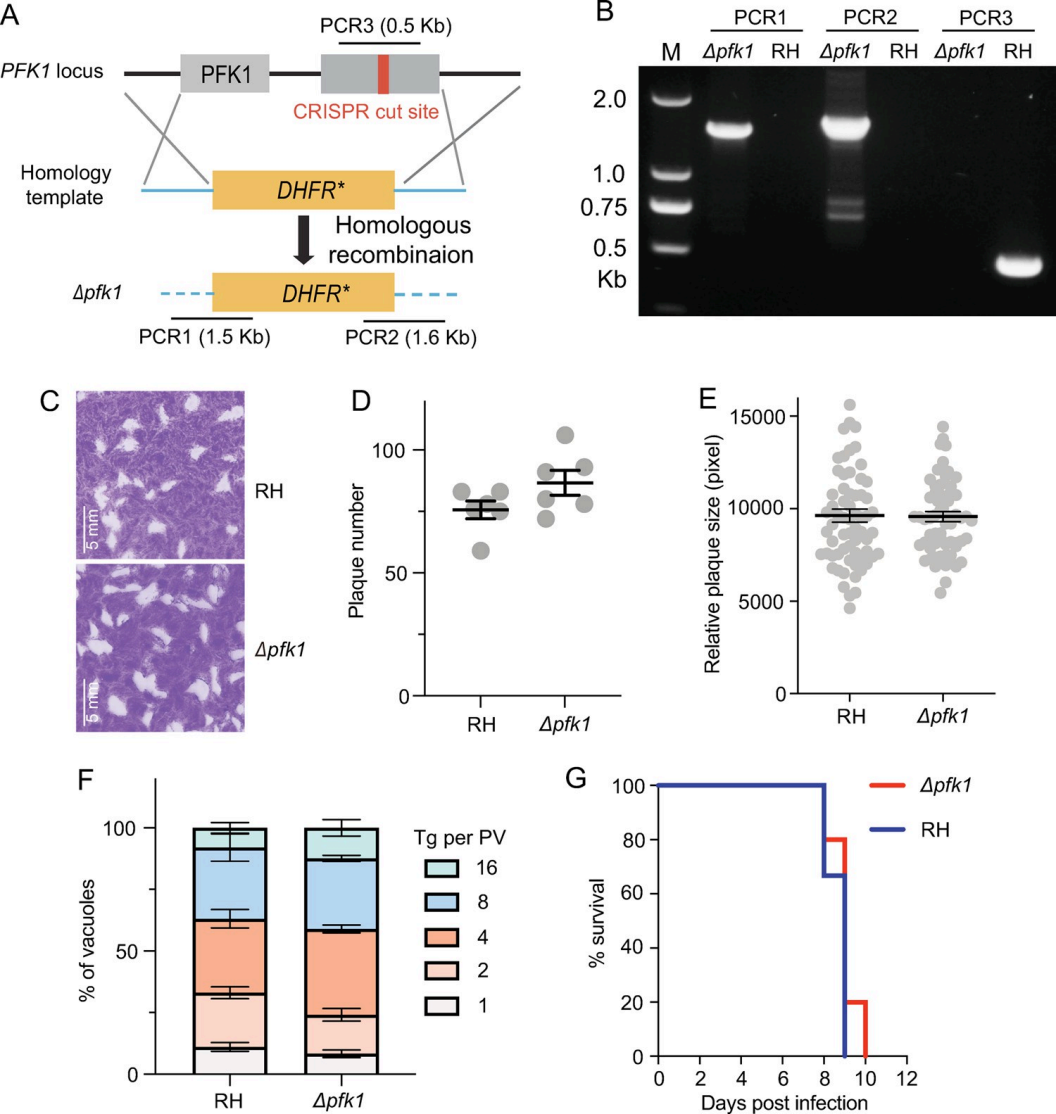
Generation and characterization of a *Toxoplasma* mutant without PFK1. **A**: Schematic diagram illustrating the replacement of *PFK1* in the RH strain with the selection marker DHFR* through CRISPR/Cas9-assisted homologous recombination. PCR1/2/3 denote the diagnostic PCRs for positive clone identification. **B**: Diagnostic PCRs on a *Δpfk1* clone. **C**: Plaque assay comparing the overall growth of the *Δpfk1* mutant to that of the wild-type strain RH. **D-E**: Numbers and sizes of plaques from C. These experiments were independently repeated twice, each with three replicates. **F**: Intracellular replication of parasites in HFF cells, as determined by the number of parasites in each parasitophorous vacuole (Tg per PV). Means ± SEM of three independent experiments. **G**: Virulence test in mice. The tachyzoites of RH or the *Δpfk1* mutant were used to infect ICR mice (100 parasites/mouse, n = 10 mice for each strain) by intraperitoneal injection, and the survival of the mice was monitored daily.

To elucidate the biological functions of the two PFKs, they were subjected to gene deletion analysis. First, CRISPR/Cas9-assisted gene replacement was used to knock out *PFK1* in the type I strain RH (Fig 2A). *Δpfk1* mutants in which the endogenous *PFK1* was replaced by the pyrimethamine resistance marker DHFR* could be readily obtained, as determined by diagnostic PCRs (Fig 2A and 2B). *PFK1* deletion did not affect the growth of tachyzoites, because the ability of the *Δpfk1* mutants to form plaques in host cell monolayers was indistinguishable from that of the parental strain (Fig 2C–2E). Similarly, intracellular replication experiments showed that the *Δpfk1* mutants proliferated as fast as the wild-type strain (Fig 2F). When used to infect mice, the *Δpfk1* mutants also displayed similar levels of virulence as the RH parental strain (Fig 2G). Taken together, these results demonstrate that the ATP-dependent PFK1 is dispensable for parasite survival, growth and virulence in *Toxoplasma*.

### The PPi-dependent PFK is essential for parasite growth both *in vitro* and *in vivo*

**Fig 3:**
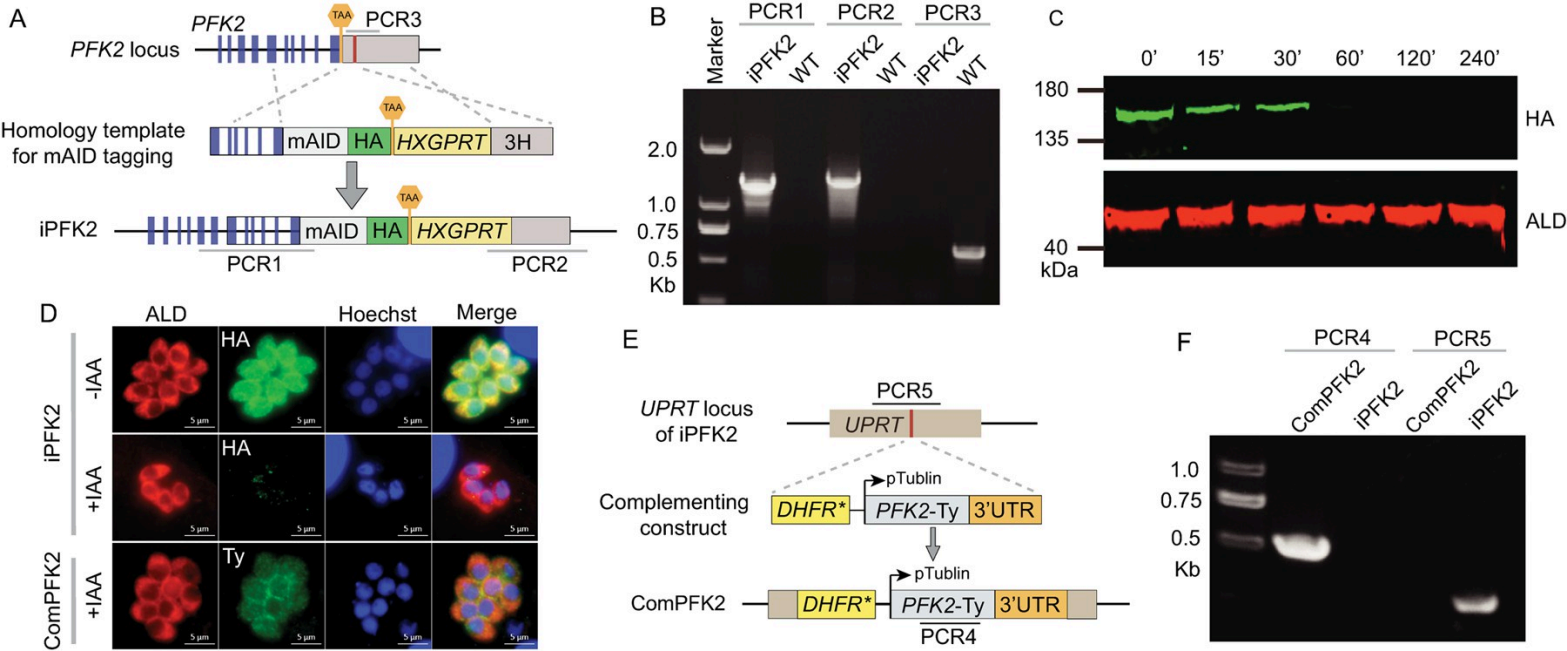
Construction of a *PFK2* depletion strain and a corresponding complementation strain. **A**: The strategy used to construct the conditional depletion strain iPFK2, which had a mAID-HA tag fused to its C-terminus at its endogenous locus. The red bar indicates the CRISPR targeting site. **B**: Diagnostic PCRs of an iPFK2 clone. The WT is the parental strain RH/Tir1. **C**: Western blots checking the degradation of PFK2 over time (from 0 to 240 minutes) in the iPFK2 strain after IAA treatment. PFK2 was detected by an HA antibody, whereas ALD was included as a loading control. **D**: Immunofluorescent staining examining the expression of PFK2 in the depletion and complementation strains. **E**: Diagram showing the strategy to construct the PFK2 complementation strain ComPFK2, which had a second copy of PFK2 expressed from the *UPRT* locus. The red bar indicates the CRISPR targeting site. **F**: Diagnostic PCRs of a ComPFK2 clone.

Using the same strategy as above to directly delete *PFK2* failed, implying that PFK2 might be critical for parasite growth. Alternatively, an auxin-induced degradation (AID) approach was used to conditionally deplete the protein level of PFK2. For this purpose, the mini AID (mAID) tag that delivers the target protein for auxin-dependent degradation was added to the C-terminus of PFK2 at the endogenous locus of a Tir1-expressing strain (Fig 3A). Diagnostic PCR was used to identify the tagged clones (iPFK2) (Fig 3B). The addition of indole-3-acetic acid (IAA, a naturally occurring auxin) rapidly reduced the protein level of PFK2 (Fig 3C and 3D). Sixty minutes after IAA treatment, PFK2 was almost undetectable by Western blotting using an antibody against the HA epitope that was incorporated into the mAID tag (Fig 3C). To assist the subsequent phenotypic analyses, we also complemented the iPFK2 strain by inserting a PFK2-Ty-expressing cassette into its *UPRT* locus (Fig 3E). A diagnostic PCR confirmed the correct integration of the inserted fragment in the complemented strain (comPFK2), and immunofluorescent staining demonstrated the expression of PFK2-Ty in the cytosol (Fig 3F and 3D).

**Fig 4:**
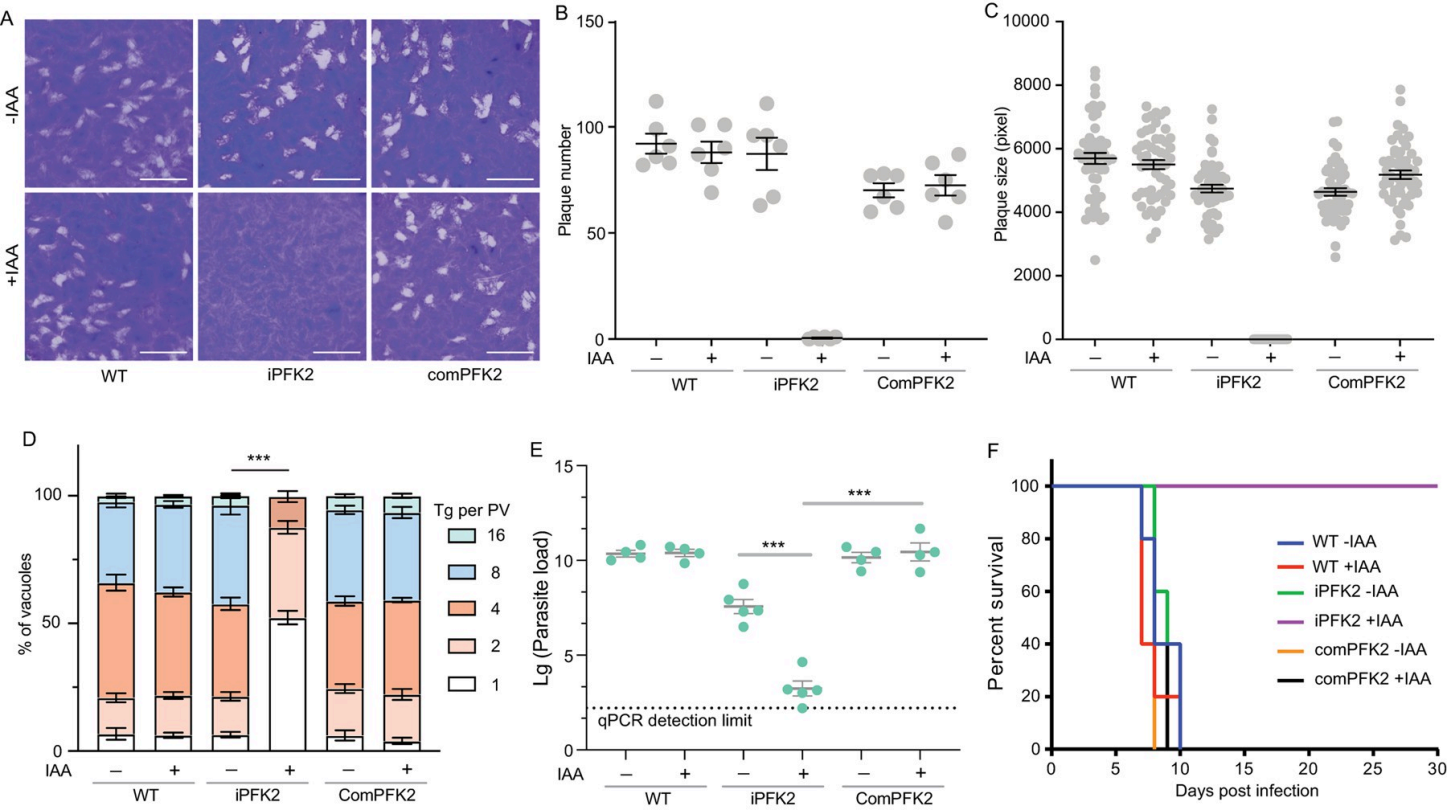
PFK2 is essential for tachyzoite growth. **A**: Plaque assay of the indicated strains in the presence or absence of IAA. **B-C**: Numbers and sizes of plaques from A. The experiments were repeated twice independently, each with three replicates. **D**: Intracellular replication of parasites in HFF cells. The means ± SEM of three independent experiments. ***p ≤ 0.001, two-way ANOVA. **E**: Parasite burden in peritoneal fluids of mice 5 days post infection, n = 4 or 5. ***p < 0.001, unpaired student’s t-test. **F**: Survival of mice treated with or without IAA after being infected with indicated strains.

To assess the role of PFK2 in parasite growth, a plaque assay was performed in the presence or absence of IAA using the iPFK2 strain. IAA treatment did not affect the growth of the wild-type strain (Tir1^+^) but completely inhibited the growth of the iPFK2 mutant, because no visible plaques were formed in the latter case (Fig 4A–4C). PFK2 complementation fully restored the growth defect, suggesting that it is responsible for this phenotypic change (Fig 4A–4C). Intracellular replication assays indicated very similar results, with the PFK2-depleted mutants proliferating much slower than the PFK2-expressing parasites (Fig 4D). Together, these data demonstrate that PFK2 has a critical role in supporting tachyzoite growth, which is consistent with the fact that the majority of tachyzoite phosphofructokinases are from PPi-PFK.

The growth of mutants lacking certain glycolytic enzymes, such as aldolase, is inhibited by glucose because of the accumulation of toxic intermediates [42]. To check whether this accumulation was the reason for the poor growth of the PFK2 depletion mutants, they were cultured in glucose-free medium, and their growth was monitored. Unlike the aldolase-deficient mutants, glucose removal did not improve the growth of the PFK2-depletion mutants. To further rule out the toxic effects of glucose, the first glycolytic enzyme, hexokinase, was deleted from the iPFK2 mutant to generate iPFK2-*Δhk* (S3A and S3B Fig). Again, IAA treatment efficiently blocked the growth and replication of the iPFK2-*Δhk* double mutant (S3C and S3D Fig), further confirming that the growth defect of the PFK2-deficient parasites was not caused by the buildup of toxic glycolytic intermediates.

To assess the role of PFK2 during parasite infection of hosts, a mouse infection model was used to determine the propagation and virulence of parasites *in vivo*. First, 10^4^ purified tachyzoites of the wildtype, iPFK2 or comPFK2 were used to infect ICR mice by intraperitoneal injection. Then the mice were treated with or without IAA that was added to drinking water. Five days after infection, the parasite loads in peritoneal were determined by quantitative PCR (qPCR). IAA treatment had little effect on parasite loads of the wildtype and comPFK2 strains, which reached 10^10^ per mouse (Fig 4E). In contrast, IAA treatment reduced the parasite loads of the iPFK2 strain to the lower sensitivity limit of qPCR and was almost 10^4^ times less than the same strain without IAA treatment (Fig 4E). These results demonstrated that PFK2 is required for parasite proliferation *in vivo*. As such, in the presence of IAA, mice infected with the iPFK2 strain did not develop obvious symptoms and all survived the infection (Fig 4F). Under the same conditions, all PFK2 expressing strains (WT -/+ IAA, iPFK2 -IAA, and comPFK2 -/+ IAA) killed infected mice within 10 days (Fig 4F).

### Metabolic alteration caused by PPi-PFK depletion

**Fig 5:**
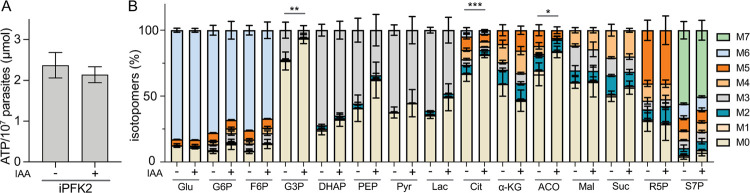
Metabolic alterations associated with PFK2 depletion. **A**: ATP levels in iPFK2 parasites with or without IAA treatment for 6 hours. **B**: Incorporation of ^13^C into the indicated metabolites in iPFK2 parasites, which were pretreated with or without IAA for 2 hours and then labeled with ^13^C_6_-glucose for 4 hours. The ^13^C inclusion in each metabolite was determined by LC-MS. M0-M7 denote the number of carbons in a given metabolite that was labeled with ^13^C. Means ± SD of three independent experiments, *P < 0.05, **P < 0.01, and ***P < 0.001, two-way ANOVA. Details of the statistical analyses are provided in S3 Table.

To understand the metabolic changes after PFK2 depletion, the cellular ATP levels were examined. Surprisingly, although IAA treatment arrested the growth of the iPFK2 mutant, it did not affect the ATP level in the parasites (Fig 5A). To check the effect of PFK2 depletion on central carbon metabolism, purified parasites were incubated in culture medium containing 8 mM ^13^C_6_-glucose for 4 hours, and then the incorporation of ^13^C into selected metabolites was analyzed by LC-MS. Compared with PFK2-expressing cells (without IAA treatment), parasites pretreated with IAA to deplete PFK2 displayed a decreased inclusion of ^13^C into a few glycolytic and TCA cycle intermediates, including glyceraldehyde-3-phosphate, citrate and aconitate (Fig 5B). The decreased incorporation of glucose-derived carbon into these metabolites suggests a reduced flux through glycolysis and the TCA cycle, which is consistent with a role of PFK2 in glycolysis. On the other hand, as a protein that is essential for parasite growth, the metabolic changes caused by PFK2 depletion are not as dramatic as expected, suggesting additional major roles of PFK2 besides as a glycolytic enzyme. Similar metabolic analyses were also performed with the *Δpfk1* mutant. Like PFK2 depletion, PFK1 disruption also led to decreased incorporation of ^13^C into glycolytic metabolites like pyruvate and lactate (S4 Fig), suggesting that it also has a role in glycolysis.

To determine whether the growth defect of the PFK2 depletion mutant was caused by reduced levels of its products or downstream metabolites, it was supplemented with FBP, lactate, pyruvate or acetate, and the subsequent growth was assessed by plaque assays. Interestingly, none of these compounds rescued the growth of the iPFK2 mutant in the presence of IAA (S5 Fig), although the parasites’ ability to take up exogenous lactate and acetate has been demonstrated before [34,43,44]. Moreover, FBP even inhibited the growth of parasites in the absence of IAA in a dose-dependent manner, which is consistent with the cytotoxicity of this compound [42,45,46]. These observations imply that reduced levels of glycolytic intermediates are probably not the primary cause of the growth defect in the PFK2 depletion mutants, again suggesting additional roles of PFK2.

### PPi-PFK depletion results in increased levels of cellular PPi and decreased rates of nascent protein synthesis

**Fig 6:**
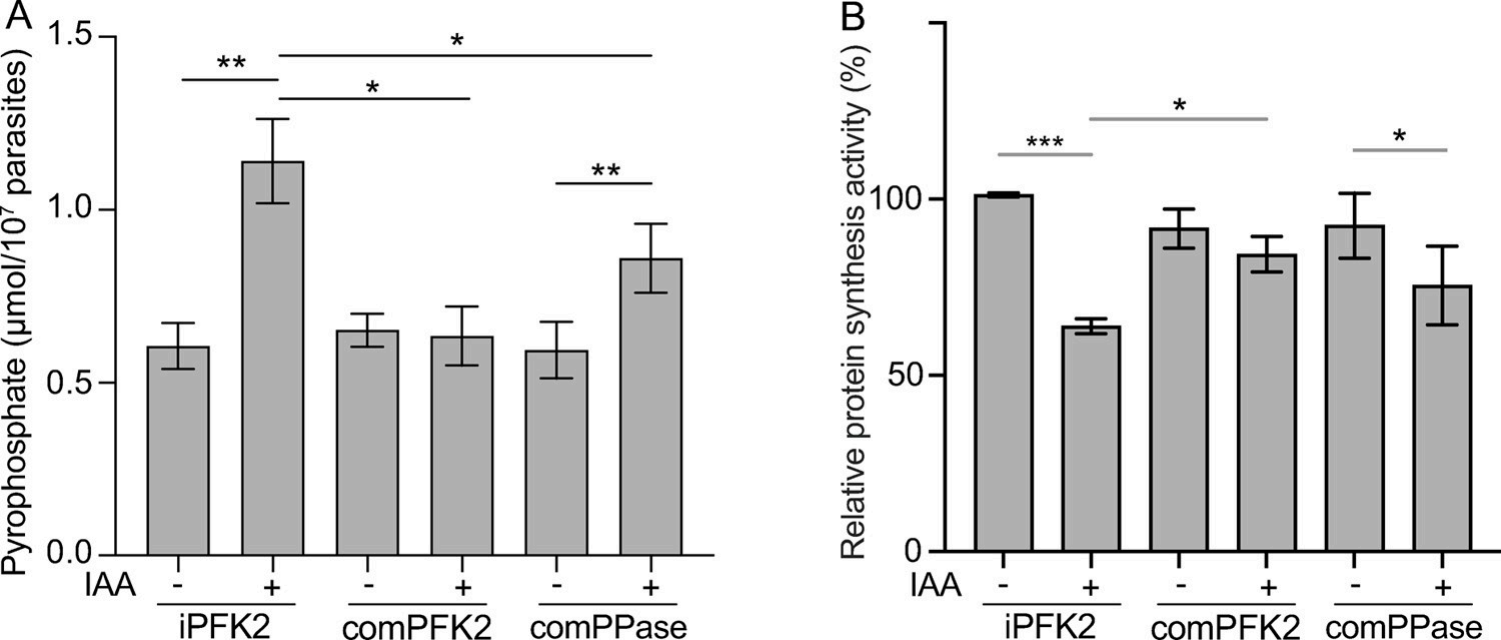
PFK2 depletion causes PPi accumulation and reduced protein synthesis. **A**: The pyrophosphate concentration in the indicated parasites with or without IAA treatment for 6 hours. Means ± SEM of four independent experiments, *P < 0.05, **P < 0.01, paired Student’s t test. **B**: Nascent protein synthesis rates in the indicated strains with or without 6 hours of IAA pretreatment. Means ± SEM of five independent experiments, *P < 0.05, ***P < 0.001, paired Student’s t test. Details for the statistical analyses are provided in S3 Table.

Since PPi-PFK also catalyzes the conversion of PPi to Pi, we sought to determine whether PFK2 depletion altered the PPi level in parasites. To this end, PPi was extracted from parasite lysate and quantified using a fluorogenic pyrophosphate sensor. PFK2 depletion by IAA treatment indeed caused a significant increase in cellular PPi, which was almost twice of that in the wild-type parasites (Fig 6A). Because high levels of PPi were thought to be deleterious to synthetic reactions that generate PPi, we looked to see whether increased PPi levels in the PFK2 depletion mutant affected biomass synthesis, using nascent protein synthesis as an example. When an alkyne moiety containing analog of methionine, L-homopropargylglycine (HPG), was added to the culture medium to probe nascent protein synthesis through a "click chemistry" method, the PFK2 depletion mutant displayed a 40% reduction in protein synthesis compared to the parental strain (Fig 6B).

### Overexpression of a soluble PPase partially rescues the growth of the PFK2 depletion mutant

**Fig 7:**
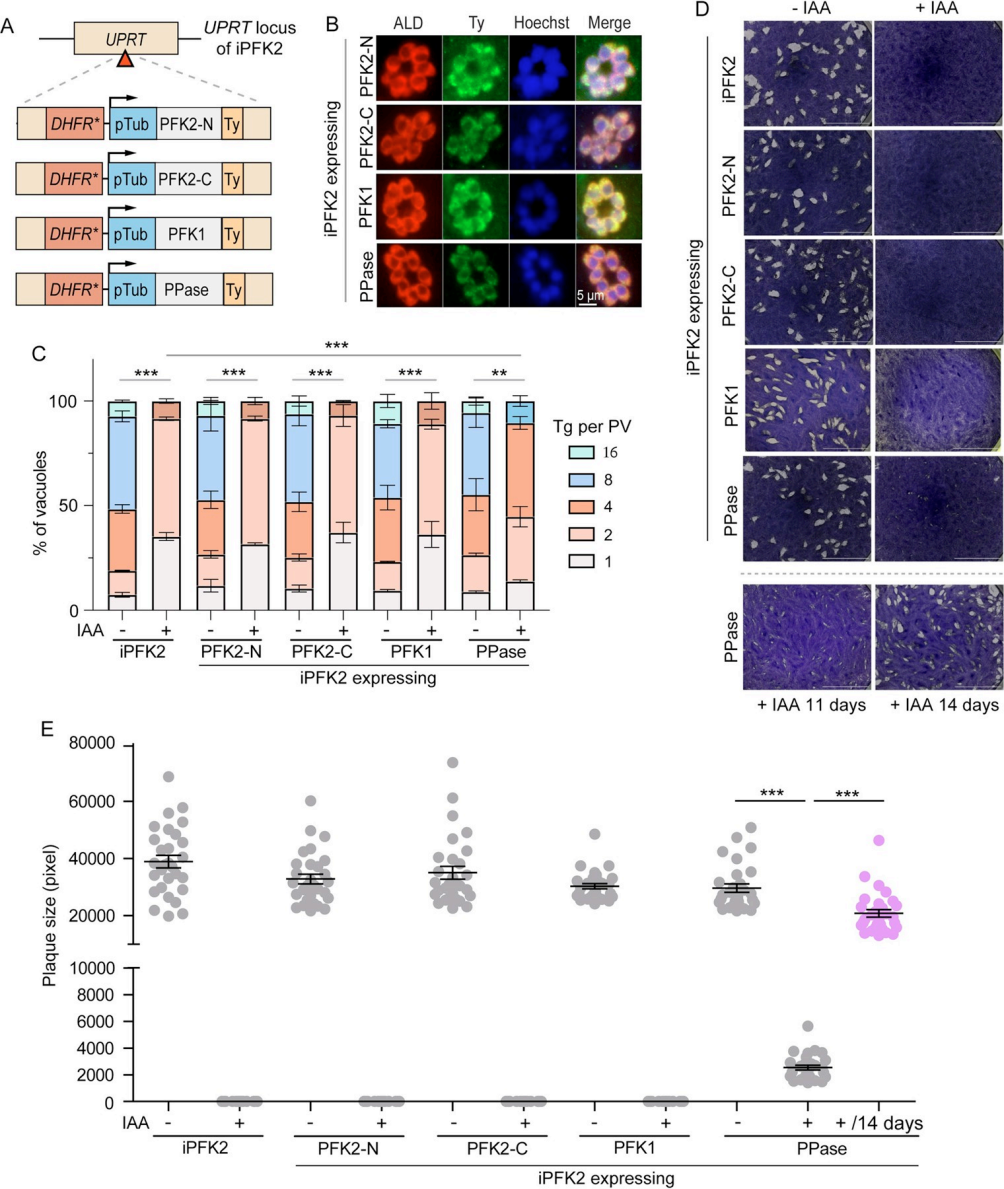
Overexpressing soluble PPase in *Toxoplasma* partially rescues the growth defect of PFK2-depletion mutants. **A**: Diagram illustrating the expression of Ppase, PFK1 or the N- or C-terminal parts of PFK2 in iPFK2 mutants from the UPRT locus. **B**: Immunofluorescence staining showing the expression of the introduced genes, as probed by the Ty tag. **C**: Intracellular replication of the indicated strains in the presence or absence of IAA. Means ± SEM of three independent experiments, **P < 0.*01*, ***P < 0.*001*, two-way ANOVA. **D**: Plaque assays for the indicated strains grown with or without IAA for 8 days or the iPFK2/PPase strain grown in the presence of IAA for 11 or 14 days. **E**: The sizes of plaques from D. ***P < 0.001, student’s t test.

The above results demonstrated that PFK2 depletion led to an excessive accumulation of PPi and decreased protein synthesis. To check whether the insufficient hydrolysis of PPi caused the growth defects of the PFK2 depletion mutants, the cytosolic PPase of *Toxoplasma* was overexpressed in the iPFK2 mutant. Transgenic PPase was inserted into the *UPRT* locus, and its expression was driven by a Tub promoter (Fig 7A). Using this strategy, transgenic PPase was expressed well, as determined by IF staining of the Ty tag that was fused to its C-terminus (Fig 7A and 7B). As shown previously, the cellular level of PPase was critical for optimal parasite growth, since its overexpression could have had an inhibitory effect [37]. In our case, the growth and replication of the iPFK2/PPase strain were indistinguishable from that of iPFK2 in the absence of IAA (Fig 7C and 7D), suggesting that the expression of the transgenic PPase was not at a level that could cause toxicity. Interestingly, when IAA was added to deplete PFK2, the iPFK2/PPase strain was able to form small plaques in a regular 8-day plaque assay (Fig 7D). By contrast, the iPFK2 mutant was not able to form any visible plaques. In addition, if the plaquing time was extended to 11 to 14 days, the iPFK2/PPase strain could form much larger plaques in the presence of IAA (Fig 7D and 7E). An intracellular replication assay also showed that extra PPase could improve the proliferation of the PFK2-depletion mutants (Fig 7C). To understand the underlying mechanism, the cellular PPi levels and protein synthesis rates were compared. The expression of extra PPase indeed reduced the PPi levels in the parasites (Fig 6A). In addition, it also increased the rate of nascent protein synthesis (Fig 6B). These results suggest that the ectopic expression of soluble PPase was able to rescue the growth of the PFK2-depletion mutants partially, likely by reducing the PPi level and increasing protein synthesis.

### Both the N- and C-terminal phosphofructokinase domains of PFK2 are required for function

Motif structure analysis suggests that PFK2 has two phosphofructokinase domains at the N- and C-terminal parts, respectively, although the C-terminal domain lacks enzymatic activity (Fig 1E). To estimate the contribution of each of these two domains (PFK2-N and PFK2-C) to the function of PFK2, they were used to complement the iPFK2 mutant individually (Fig 7A). When they were expressed at similar levels (Fig 7B), neither one was able to rescue the growth or intracellular replication of iPFK2 in the presence of IAA (Fig 7C–7E). These results indicate that both domains are essential for the function of PFK2.

Using a similar set of experiments, we tested whether the overexpression of ATP-PFK (PFK1) was able to improve the growth of PFK2-depletion mutants. When expressed at the *UPRT* locus of iPFK2, similar to other complements described above (Fig 7A), PFK1 was well expressed (Fig 7B). However, it was not able to restore the growth or replication of iPFK2 in the presence of IAA (Fig 7C–7E), further suggesting that the lack of phosphofructokinase activity that converts F6P to FBP is not the primary cause of the growth defect in PFK2-depleted parasites, which otherwise should be rescued by PFK1 overexpression.

## Discussion

**Fig 8:**
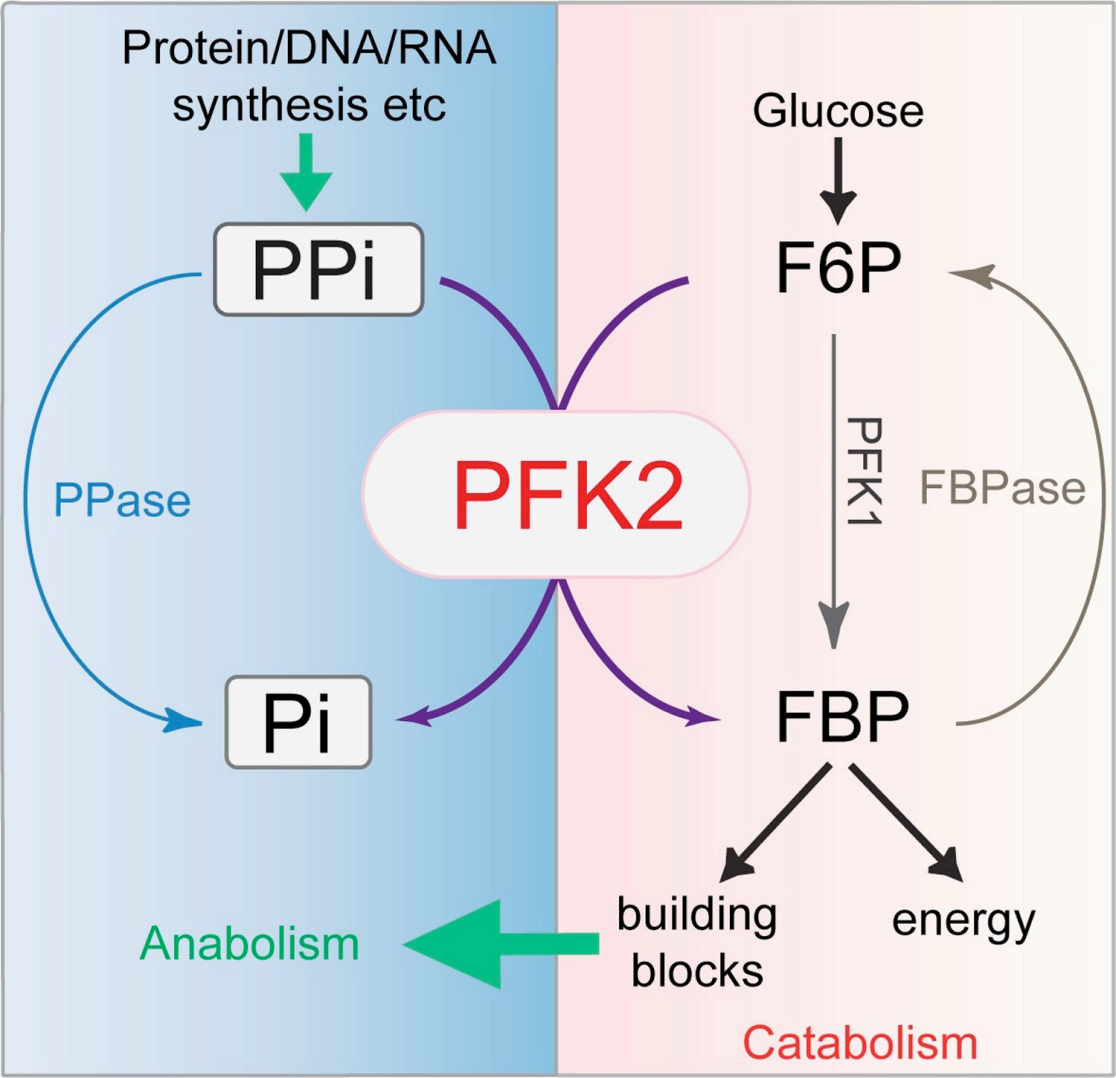
A model for PFK2 in maintaining PPi homeostasis and balanced catabolism and anabolism in *Toxoplasma*. The biosynthesis of macromolecules such as proteins, DNA and RNA generates PPi, which is then converted to Pi by PPase and the PPi-dependent phosphofructokinase PFK2. The hydrolysis of PPi by PFK2 is coupled to the phosphorylation of F6P to FBP, the committed step of glycolysis. Through the activity of PFK2, PPi is hydrolyzed, which otherwise would inhibit biosynthetic reactions. In addition, glycolysis is activated to produce more building blocks for macromolecule synthesis as well as energy. As such, PFK2 serves as a key factor in coordinating the parasites’ catabolic and anabolic activities. PFK2-deficient parasites suffer from increased levels of PPi, reduced macromolecule biosynthesis and decreased glycolysis. PFK1 may be able to rescue in part the defects in glycolysis caused by PFK2 depletion, but it would not be able to reverse the defects in PPi homeostasis and macromolecule synthesis.

*T*. *gondii* expresses two active phosphofructokinases in the cytosol. They have distinct substrate specificity in terms of phosphoryl donor preference. PFK1 uses ATP, whereas PFK2 uses PPi as phosphoryl donors and energy sources, respectively. Through genetic approaches, we showed that ATP-dependent PFK1 is dispensable for tachyzoite growth or virulence, whereas the PPi-dependent PFK2 is essential. This finding is consistent with the fact that PPi-PFK is responsible for over 94% of the PFK activity in tachyzoite extracts [38]. Metabolic analyses suggested that PFK2-depleted parasites had normal levels of ATP and moderate decrease in the flux of glycolysis and the TCA cycle. However, PFK2 depletion leads to PPi accumulation and reduced protein synthesis. More importantly, overexpressing the soluble PPase that hydrolyzes the excess PPi in the PFK2-depleted parasites significantly rescued its growth defects. The overexpression of the ATP-dependent phosphofructokinase PFK1 in the same mutant did not rescue these growth defects. These results indicate that PPi mis-regulation is the primary cause of the growth defect in the PFK2-deficient mutants. Taken together, our data suggest a critical role for PFK2 in maintaining pyrophosphate homeostasis in *Toxoplasma*. PPi is the byproduct of a number of biosynthetic reactions, such as the synthesis of proteins, DNA and RNAs. Its accumulation does not favor the biosynthesis of these macromolecules and is toxic to cells. PFK2 uses PPi consumption to power the conversion of F6P to FBP, the committed step of glycolysis. PFK2 thus has a pivotal role in coordinating the activities of catabolism and anabolism in parasites (Fig 8).

Cellular PPi level is strictly regulated and almost all living organisms express pyrophosphatases to convert PPi to Pi. Defects in pyrophosphatase activities are often lethal, although the detailed mechanisms are not fully appreciated yet. In yeasts, conditional repression of *IPP1*, the gene encoding a soluble pyrophosphatase, alters cell cycle progression and arrests yeast growth [47]. *Toxoplasma* also encodes a soluble pyrophosphatase (PPase) and its overexpression is detrimental to parasite growth [37]. The extremely low phenotype score (-4.64) (ToxoDB, [48]) of the gene encoding the cytosolic PPase implies that its deficiency would also cause parasite growth arrest. But the exact physiological function of *Toxoplasma* PPase requires further investigation. In addition to PPase, the PPi-dependent PFK2 in *T*. *gondii* also converts PPi to Pi and is involved in parasite PPi homeostasis, as our results demonstrated. However, unlike the suppression of *IPP1* in yeasts, depletion of PFK2 in *T*. *gondii* did not alter the cell cycle profile when the parasites were examined by propidium iodide staining that determined the DNA contents (S6 Fig). This result was initially surprising to us since PFK2 depletion led to increased PPi level (Fig 6A), which was theoretically unfavorable for biosynthetic reactions like protein and DNA synthesis. We did observe that the nascent protein synthesis activity in the parasites was reduced by 40% upon PFK2 depletion (Fig 6B). As such, a delay or inhibition of DNA synthesis was expected in this mutant. Yet, propidium iodide staining and subsequent flow cytometry analyses did not find significant defect in DNA replication. This may be due to that DNA synthesis is less sensitive to PPi than protein synthesis. For instance, when we do PCR reactions in test tubes, we never add any pyrophosphatase to remove PPi. Or, as a recent study reported, PPi hydrolysis may be an intrinsic step of the DNA synthesis reaction catalyzed by DNA polymerases [49]. Nonetheless, neither explanation can explain the discrepancy in cell cycle alteration between *IPP1* suppression in yeasts and PFK2 depletion in *Toxoplasma*. PPase and PPi-PFKs may work differently although both can consume PPi. Indeed, both the cytosolic PPase and PPi-PFK (PFK2) are critical, instead of redundant or synthetic lethal, for the growth of tachyzoites. On the other hand, the partial rescue of PFK2 mutants by PPase suggests that they do have overlapping functions. Interestingly, we showed that PFK2-N had full PPi-PFK activity as the full-length PFK2 enzyme (Fig 1E). Yet it was not able to complement the PFK2 depletion mutant (Fig 7), suggesting additional roles of PFK2 besides the PPi-PFK enzymatic activity and they deserve further investigations.

The function and regulation of glycolysis in the obligate intracellular parasite *T*. *gondii* are complicated, partly due to its parasitic lifestyle in diverse hosts and host cell types. This parasite exhibits great metabolic flexibility and is able to use glucose, glutamine and even lactate and alanine as carbon sources. As a consequence, the disruption of some of the proteins involved in glucose utilization through glycolysis had only a mild effect on parasite growth, such as the primary glucose importer GT1 and the first enzyme in glycolysis HK [31,32]. Notably, the gluconeogenic enzyme FBPase2 was shown to be essential for tachyzoite growth, even in the presence of glucose [35]. This finding is unexpected, and it was proposed that futile cycling between FBP and F6P is critical for parasite growth, because it allows for rapid adaptation to changing environments. PFK2 catalyzes the reverse reaction of FBPase2. Therefore, it is not surprising that PFK2 is essential for parasite survival. However, the involvement of PFK2 in the cycling between FBP and F6P suggests that it is not truly futile. It actually results in a net conversion of PPi to Pi. As explained above, due to the key role of PPi homeostasis in coordinating catabolic and anabolic metabolism, FBPase2 and FBP/F6P cycling may not only allow for quick adaptation but may also have key regulatory roles in metabolic homeostasis. Consistent with this notion, FBPase2-deficient mutants also had reduced rates of amylopectin and GPI biosynthesis [35].

*Toxoplasma* is one of the few organisms that have a full set of enzymes involved in PPi hydrolysis and F6P/FBP interconversion, in addition to plants and some bacteria. These include ATP-PFK, PPi-PFK, FBPase, soluble and membrane-associated PPases (S7 Fig). Among these, FBPase2 and soluble PPase were shown to be critical for parasite growth [35,37]. Here, we show that PPi-PFK is also essential, suggesting that PPi homeostasis and the coupled F6P/FBP conversion are indeed key points for regulation to ensure the optimal growth of parasites. While *T*. *gondii* expresses both ATP-PFK (PFK1) and PPi-PFK (PFK2), why is PPi-PFK more active and more important? This may be in part because PPi-PFK uses the energy from PPi consumption to drive FBP production, which is believed to be more energy efficient [47]. This property can be advantageous for parasitism. More importantly, we think that the activity of PPi-PFK links the rate of glycolysis (catabolism) to that of macromolecule synthesis (anabolism, through PPi) (Fig 8). Therefore, it can fine tune the activities of these two metabolic directions and increase the utilization efficiency of the limited nutrients taken up from host cells, maximizing the chance of successful parasitism.

## Materials and methods

### Ethics statement

Seven-week-old female ICR mice were purchased from the Hubei provincial Center of Disease Control and Prevention and maintained under specific pathogen-free conditions, according to the guidelines specified by the Administration of Affairs Concerning Experimental Animals in Huazhong Agricultural University. All animal experiments were approved by the ethical committee of Huazhong Agricultural University (permit number HZAUMO-2019-058).

### Parasite strains and growth conditions

All the genetically modified strains used in this study were generated from the parental strains RH *Δhxgprt* (RH) or RH *Δhxgprt* Tir1 (RH-Tir1). All the parasites were cultivated in HFF (ATCC, VA, USA) monolayers in DMEM (Dulbecco’s modified Eagle’s medium) supplemented with 2% fetal bovine serum, 10 U/ml penicillin, 100 μg/ml streptomycin and 10 mM glutamine.

### Chemicals and antibodies

IAA was purchased from Sigma-Aldrich. Anti-Ty (mouse), anti-TgSAG1 (mouse), and anti-TgALD (rabbit) antibodies were provided by Prof. David Sibley (Washington University in St. Louis, USA). Anti-HA (mouse) was purchased from MBL (Medical & Biological Laboratories Co., Japan). Alexa Fluor-conjugated goat anti-mouse and goat anti-rabbit secondary antibodies were purchased from Thermo Fisher Scientific. IRDye 800CW-conjugated goat anti-mouse and IRDye 680RD-conjugated goat anti-rabbit secondary antibodies (Life Technologies, USA) were used for Western blots.

### Construction of plasmids and transgenic parasites

The plasmids and primers used in this study are listed in S1 and S2 Tables, respectively. Locus-specific CRISPR constructs were generated by replacing the UPRT-targeting gRNA in pSAG1-Cas9-U6-sgUPRT with gene-specific gRNAs, following the protocol described elsewhere [50,51]. Other plasmids were constructed using multi-fragment cloning with the ClonExpress one-step cloning kit (Vazyme Biotech, Nanjing, China). Details on how each plasmid or fragment was constructed or generated are provided in S1 Table.

To construct the iPFK2 mutant, the PFK2-mAID-HXGPRT amplicon (derived from the plasmid pAID::PFK2-HA, S1 Table) was co-transfected with pSAG1-Cas9- sgPFK2-cKO into the RH *Δhxgprt* Tir1 strain. Transfectants were selected with 25 μg/ml mycophenolic acid and 50 μg/ml xanthine (Sigma Aldrich, St. Louis, MO, USA), cloned by limiting dilution, and identified by diagnostic PCRs (primers listed in S2 Table). PFK2 degradation in the iPFK2 strain was induced by adding indole-3-acetic acid (IAA) (Sigma Aldrich, St. Louis, MO, USA) to culture medium to a final concentration of 500 μM. The complementation strain ComPFK2, as well as the strains expressing PFK2N, PFK2C, PFK1 or PPase in iPFK2 were generated by inserting the corresponding amplicons (derived from the plasmids listed in S1 Table) into the *UPRT* locus of iPFK2, through co-transfection with pSAG1-Cas9-sgUPRT. Transfectants were selected with 1 μM pyrimethamine (Sigma Aldrich, St. Louis, MO, USA) and the monoclonal strains were identified by diagnostic PCRs and IFA before use.

### Phenotypic analysis of parasites

Standard plaque assays and intracellular replication assays were used to assess the overall growth and proliferation of *T*. *gondii* parasites, following previously described protocols [51–53]. To test the virulence of the *Δpfk1* mutant and its corresponding wildtype strain, freshly egressed tachyzoites were purified and used to infect mice by intraperitoneal injection (10 mice per strain, 100 parasites per mouse). The survival of the mice was monitored daily.

To examine the proliferation of the PFK2 depleted parasites *in vivo*, ICR mice were infected with 10^4^ tachyzoites of designated strains by intraperitoneal injection (four to five mice per strain per treatment). Five days after infection, the peritoneal fluids of the infected mice were collected, and genomic DNA was extracted from them using the TIANamp Genomic DNA Kit (Tiangen Biotechnology Co. Ltd., China). To generate a standard curve for parasite quantitation, 0, 10, 10^2^, 10^3^, 10^4^, 10^5^, 10^6^, and 10^7^ tachyzoites of the RH-Tir1 strain were mixed with 2 ml peritoneal fluid derived from non-infected mice and genomic DNA was extracted respectively. The parasite loads in the peritoneal fluids were determined by quantitative PCR (qPCR) amplification of the *T*. *gondii* GAPDH1 gene (primers listed in S2 Table) using the Power SYBR Green PCR Master Mix (Toyobo Co., Ltd., Osaka, Japan), according to the manufacturer’s instructions. PCR reactions were performed on the ABI ViiA 7 system (Life Technologies, Inc., USA). The C_T_ value of each sample was used to calculate the corresponding parasite load, according to the standard curve generated. Each sample had three technical repeats for the qPCR.

The virulence of the PFK2 depletion mutant *in vivo* was tested following a previously described protocol [54]. Briefly, the iPFK2 strain and corresponding control lines were used to infect seven-week-old female ICR mice (100 tachyzoites per mouse, 5 mice per strain per condition). Then the mice were treated with or without IAA (3-indoleacetic acid, Sigma-Aldrich, USA) for 30 days. The IAA treatment groups were supplied with drinking water containing 1.34 mg/ml IAA, 3 mM NaOH, 5% sucrose (w/v), cherry flavored tablets (4 pill/L, Yangshengtang Pharmaceutical Co., LTD, China), pH = 7.4 (adjusted with NaOH). The control groups received the same drinking water but lacking IAA.

### Bioinformatics analysis

ClustalX 2.1 was used for sequence alignments. The sequences were downloaded from the UniProt and ToxoDB websites. The access numbers for the corresponding proteins are as follows: (TgPFK1,ToxoDB gene ID: TGGT1_240890; ChPFK, *Cryptosporidium hominis* PFK, UniProtKB ID: A0A0S4TBK3_CRYHO; TbPFK, *Trypanosoma brucei brucei* PFK, UniProtKB ID: PFKA_TRYBB; LdPFK, *Leishmania donovani* PFK, UniProtKB ID: PFKA_LEIDO; CePFK, *Caenorhabditis elegans* PFK, UniProtKB ID: PFKA1_CAEEL; EhPFK, *Entamoeba histolytica* PFK, UniProtKB ID: PFKA_ENTHI; EcPFK, *Escherichia coli* (strain K12) PFK, UniProtKB ID: PFKA_ECOLI; AtPFK, *Arabidopsis thaliana* PFK UniProtKB ID: PFKA3_ARATH; HsPFK, *Homo sapiens* PFK, UniProtKB ID: PFKAP_HUMAN; SsPFK, *Sus scrofa* PFK, UniProtKB ID: PFKAM_PIG); ScPFK, *Saccharomyces cerevisiae* PFK (strain ATCC 204508/S288c), UniProtKB ID: PFKA1_YEAST; SaPFK, *Staphylococcus aureus* PFK, UniProtKB ID: PFKA_STAA8; EaPFK, *Eimeria acervulina* PFK, UniProtKB ID: U6G9T9_EIMAC; TaPFK, *Triticum aestivum* PFK, UniProtKB ID: A0A1D5UP90_WHEAT; TgPFK2, *Toxoplasma gondii* phosphofructokinase PFKII, ToxoDB gene ID:TGGT1_226960; ChPFP, *Cryptosporidium hominis* PFP, UniProtKB ID: A0A0S4TCQ1_CRYHO; EhPFP, *Entamoeba histolytica* PFP, UniProtKB ID: PFP_ENTHI; EaPFP, *Eimeria acervulina* PFP, UniProtKB ID: U6GFF7_EIMAC; Ath PFP, *Arabidopsis thaliana* PFP, UniProtKB ID: PFPB1_ARATH; TaPFP, *Triticum aestivum* PFP, UniProtKB ID: A0A1D6D1Q3_WHEAT; and StPFP, *Spirochaeta thermophila* PFP, UniProtKB ID: PFP_SPITD.

### Protein purification and enzymatic assays

The plasmids that allowed the expression of PFK1 (pESUMO-PFK1) and PFK2 (pESUMO-PFK2, pESUMO-PFK2C or pESUMO-PFK2N) were individually transformed into Transetta (DE3) cells, and the expression of recombinant proteins was induced with 1 mM IPTG. Subsequently, proteins were purified on a Ni^2+^ column, and the concentrations were determined with a BCA protein assay kit (Beyotime, China). Then, the enzymatic activities of purified proteins were tested using colorimetric PFK/PFP activity assay kits (Solarbio, China). The reactions were performed in 200 μl (ATP-PFK assay) or 500 μl (PPi-PFK assay) assay buffer containing 1.76 mg/ml protein and F6P (0, 100, 200, 500, 1000, 2000, 3000 or 4000 μM). Since the enzyme activities were eventually coupled to NADH oxidation, the OD_340_ of the reaction samples was monitored for 4 min (ATP-PFK assay) or 7.5 min (PPi-PFK assay), and the slopes of the OD_340_ curves were used to calculate the PFK1 and PFK2 activity using the following equation: V = [ΔA×V_total_ ÷ (ε×d) ×10^9^] ÷ (Cpr × Vol _sample_)) ÷T, ε = 6.22×10^3^ L/mol/cm, d = 0.2 cm, Cpr:1.76 mg/ml, Vol _sample_ = 5 μl (ATP) or 50 μl (PPi). Each assay was repeated three times independently.

### Pyrophosphate and ATP concentration measurements

To measure the cellular levels of PPi, the iPFK2 and related strains were treated with or without 500 μM IAA for 6 h immediately before natural egress. Then, freshly egressed parasites were filtered through 3 μm polycarbonate membranes to remove the host cell debris and washed 3 times in ice-cold PBS. Subsequently, 3×10^7^ parasites were suspended in 100 μl of ice-cold 0.5 M HClO_4_. After 30 min of incubation on ice, the extracts were centrifuged at 3000 g for 5 min. The supernatants were neutralized by adding 50 μl of 0.72 M KOH/0.6 M KCl. The centrifugation was repeated to remove the precipitated KClO_4,_ and the pH of the separated supernatant was adjusted to 8.0 with 0.1 M KOH. Then, the PPi level was tested using a commercial pyrophosphate assay kit (Sigma–Aldrich, USA) coupled to a fluorogenic pyrophosphate sensor.

For ATP concentration measurements, 3×10^7^ purified parasites were suspended in 150 μl lysis buffer, cooled on ice for 30 min, and then centrifuged at 12000 g for 5 min at 4°C to collect the supernatants, as previously described. The ATP levels in the supernatant were examined using a colorimetric ATP assay kit (Beyotime, China) by following the instructions from the manufacturer.

### Determining the rates of nascent protein synthesis

Nascent protein synthesis rates in parasites were determined by previously described methods [55]. Briefly, HFF cells seeded in T25 flasks were infected with the indicated parasite strains and cultured for 30 hours. Then, IAA was added to a final concentration of 500 μM to treat the parasites for 1 hour to deplete the target protein expression. Subsequently, the culture medium was changed to L-methionine-free Grace’s Insect Medium (Thermo Fisher Scientific, USA) containing 2% dialyzed FBS and 100 μM homopropargylglycine (APExBIO, USA). The parasites were labeled for 4 hours in this medium, purified by needle-induced egress and purified by 3 μm membrane filtration. The collected parasites were then washed twice with PBS, fixed with 4% paraformaldehyde, permeabilized with 0.1% Triton X-100 and blocked with 10% FBS. Then, they were subjected to a click reaction in a freshly prepared buffer containing copper sulfate (200 μM), TCEP (400 μM), and Tris (3-hydroxypropyltriazolylmethyl)-amine (TBTA, Sigma-Aldrich) (200 μM). After that, FAM azide (5-isomer) (APExBIO, USA) at a final concentration of 10 μM was added and incubated with the parasite samples at 4°C overnight in the dark. The samples were then washed with PBS, and the fluorescence intensity of each parasite cell was analyzed with a Cytoflex LX flow cytometer (Beckman Coulter, USA), for a recording of more than 10,000 parasites per sample. The data were analyzed with CytExpert software (Version 2.4.0.28, Beckman Coulter, USA).

### Metabolomic analysis

Fresh extracellular tachyzoites (3×10^7^) with or without IAA pretreatment were labeled with 8 mM [^13^C_6_]-glucose in glucose-free DMEM for 4 hours. Then, the parasites were collected, washed with PBS and lysed in 1 ml of ice-cold methyl alcohol (80%). The mixture was subjected to ultrasonication (6 cycles of 10 min, each with 1 min interval) and then centrifuged at 16000 g for 15 min at 4°C. The supernatant was dried under a nitrogen stream and then resuspended in 50 μl double distilled H_2_O. The extracted metabolites were then analyzed by LC-MS using a Dionex Ultimate 3000 UPLC system coupled to a TSQ Quantiva Ultra triple-quadrupole mass spectrometer (Thermo Fisher, CA) equipped with a heated electrospray ionization (HESI) probe. The extracts were separated with a Synergi Hydro-RP column (2.0×100 mm, 2.5 μm, Phenomenex, USA). A binary solvent system was used, in which mobile phase A consisted of 10 mM tributylamine adjusted with 15 mM acetic acid in water and mobile phase B was methanol. The analysis was performed using a 25-minute gradient from 5% to 90% mobile B. The data were acquired in selected reaction monitoring (SRM) with positive-negative ion switching mode. The resolution for Q1 and Q3 were both 0.7 FWHM. The source parameters were as follows: spray voltage: 3000 V; capillary temperature: 320°C; heater temperature: 300°C; sheath gas flow rate: 35 Arb; and auxiliary gas flow rate: 10 Arb. Data analysis and quantitation were performed in TraceFinder 3.1 software (Thermo Fisher Scientific, USA).

### Statistical analysis

Statistical analyses were performed in Prism 9 (GraphPad Software Inc., La Jolla, CA, USA) using Student’s t tests or a two-way analysis of variance, as indicated in the figure legends.

## Supporting information

S1 Fig Structure modeling of PFK1 and PFK2 to compare their ATP or PPi binding pockets. The homology-based structure modeling program SWISS-MODEL was used to predict the structure of the target proteins. The human platelet phosphofructokinase (PDB: 4XYJ) was used as a template for PFK1 modeling, whereas the *Borrelia burgdorferi* pyrophosphate-dependent phosphofructokinase (PDB: 2F48) was used for PFK2. An enlarged view of the ATP/PPi binding pockets is shown, with the critical Asp/Gly residues highlighted by colored sticks.
(TIF)

S2 Fig Localization of the two PFK proteins in *Toxoplasma* parasites as determined by epitope tagging. The endogenous gene was tagged with an smHA tag (PFK1) or a Ty tag (PFK2) at the C-terminus through CRISPR/Cas9-mediated site-specific insertion. Subsequently, the recombinant parasites were subjected to immunofluorescent staining using antibodies against the corresponding epitope tags, as well as ALD to stain the parasite cytoplasm.
(TIF)

S3 Fig Inactivation of hexokinase (HK) in the PFK2 conditional knockdown strain. **A.** Schematic illustration of deleting *HK* in iPFK2 to produce the iPFK2-*Δhk* double mutant. **B.** Diagnostic PCRs on a selected iPFK2-*Δhk* clone. **C.** Plaque assay comparing the growth of the iPFK2-*Δhk* mutant with or without IAA treatment. **D.** Comparison of the intracellular replication efficiency of the iPFK2-*Δhk* mutant treated with or without IAA. ***P < 0.001, Two-way ANOVA (n = 3).
(TIF)

S4 Fig Incorporation of ^13^C into indicated metabolites in wildtype (WT) or *Δpfk1* parasites, which was determined by LC-MS as in Fig 5B. M0-M7 denotes the number of carbons in a given metabolite that was labeled with ^13^C. Means ± SD of three independent experiments, *P < 0.*05*, **P < 0.*01*, and ***P < 0.*001*, two-way ANOVA.
(TIF)

S5 Fig Growth of the iPFK2 mutants supplemented with the indicated compounds, as determined by plaque assays in the presence or absence of IAA. (TIF)

S6 Fig Cell cycle progression of indicated parasite strains determined by propidium iodide (PI) staining. Intracellular parasites with or without IAA treatment for 12 hours were released from host cells by needle passage and then subject to PI staining and flow cytometry analysis.
(TIF)

S7 Fig Distribution of selected enzymes involved in F6P/FBP conversion and PPi consumption in diverse organisms. FBPase: fructose-1,6-bisphosphatase; mPPase: membrane-associated pyrophosphatase; sPPase: soluble pyrophosphatase.
(TIF)

S1 Table Plasmids used in this study. (DOCX)

S2 Table Primers used in this study. (DOCX)

S3 Table Statistical analysis results of datasets used in Figs 5 and 6. (XLSX)
