# Supplementary figures and images for: Essential role of pyrophosphate homeostasis mediated by the pyrophosphate-dependent phosphofructokinase in Toxoplasma gondii
Source: PLoS Pathog. 2022 Feb 1;18(2):e1010293. doi: 10.1371/journal.ppat.1010293 (PMC8836295; doi:10.1371/journal.ppat.1010293)

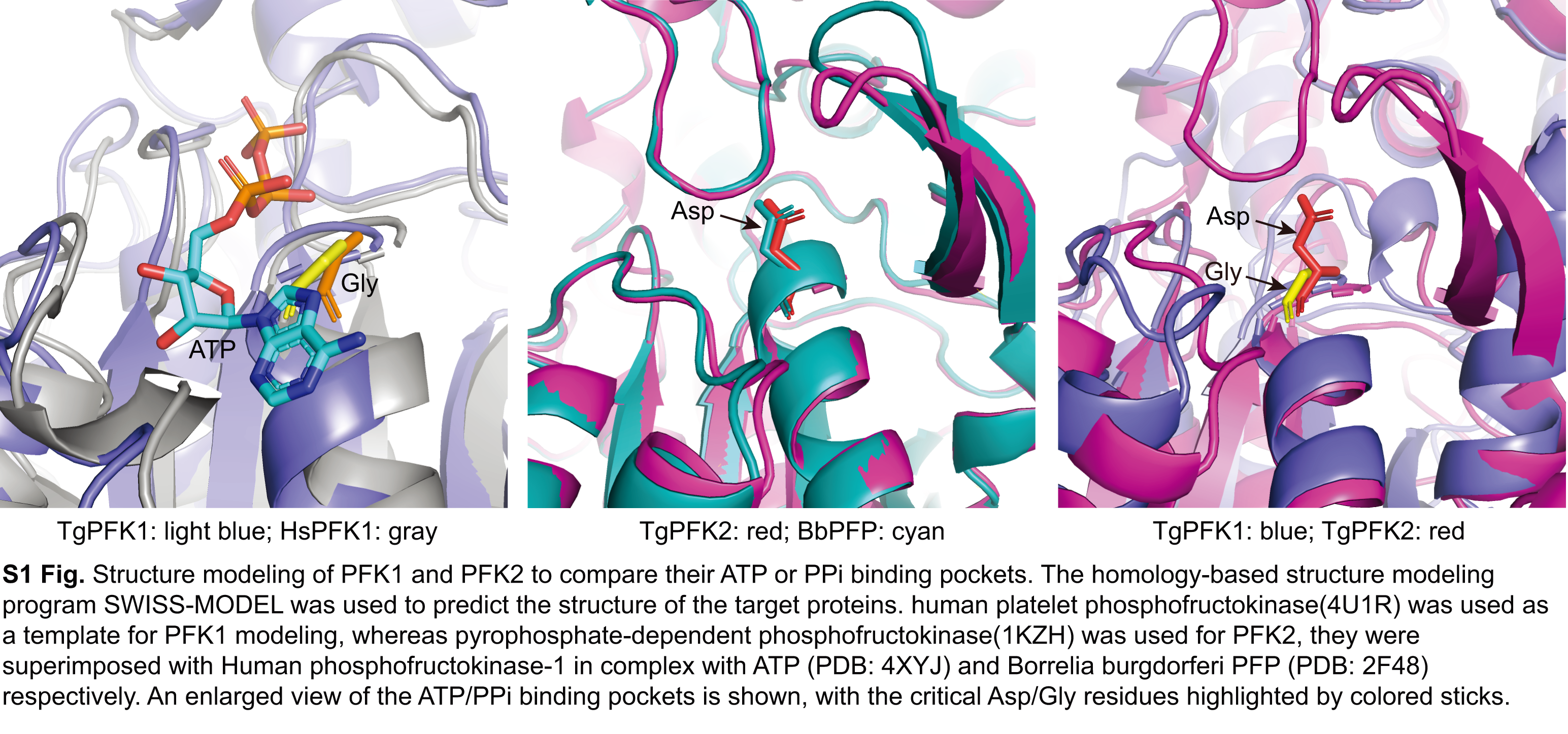

Supplement: S1 Fig — The homology-based structure modeling program SWISS-MODEL was used to predict the structure of the target proteins. The human platelet phosphofructokinase (PDB: 4XYJ) was used as a template for PFK1 modeling, whereas the Borrelia burgdorferi pyrophosphate-dependent phosphofructokinase (PDB: 2F48) was used for PFK2. An enlarged view of the ATP/PPi binding pockets is shown, with the critical Asp/Gly residues highlighted by colored sticks. (TIF) [file ppat.1010293.s001.tif]

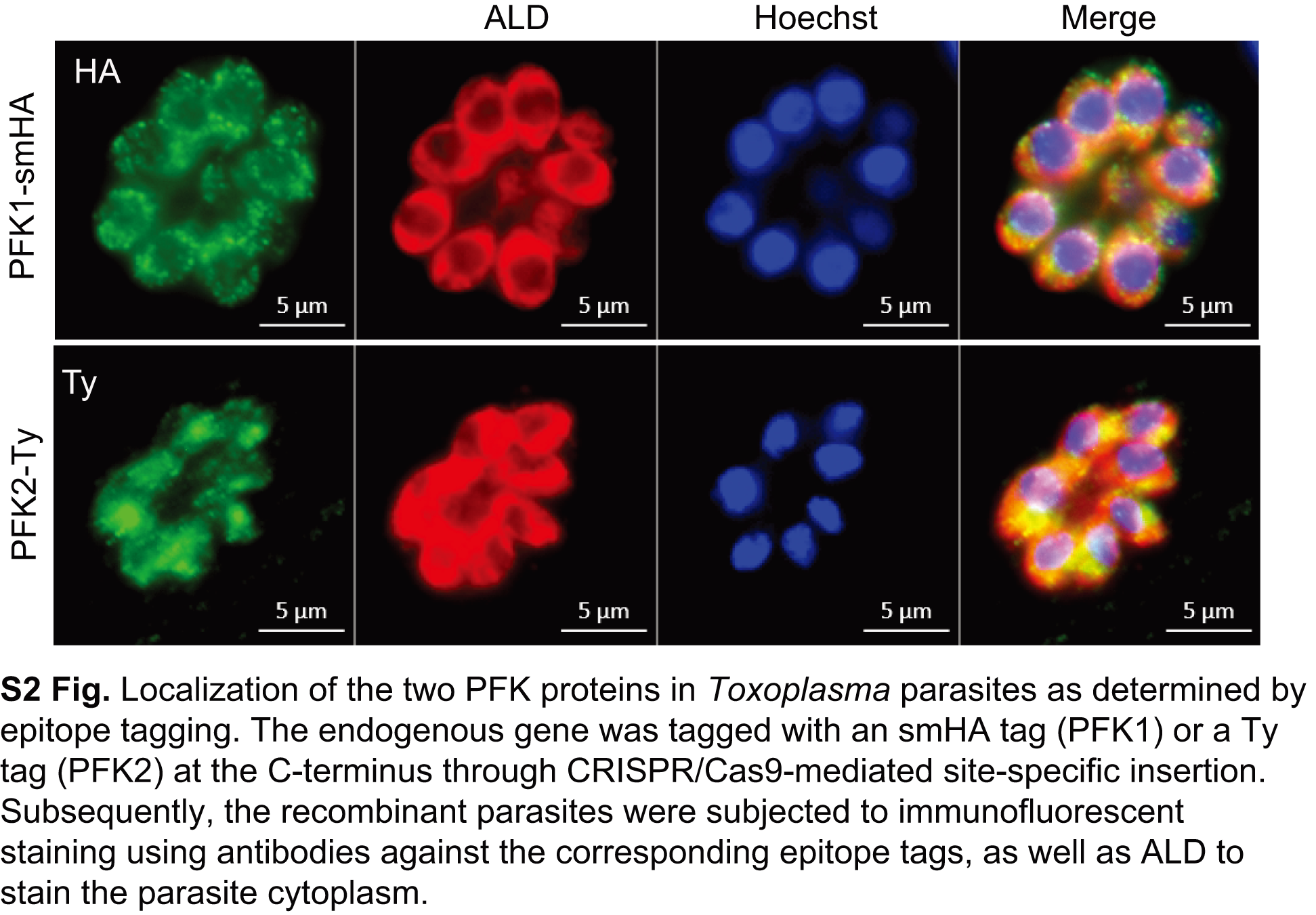

Supplement: S2 Fig — The endogenous gene was tagged with an smHA tag (PFK1) or a Ty tag (PFK2) at the C-terminus through CRISPR/Cas9-mediated site-specific insertion. Subsequently, the recombinant parasites were subjected to immunofluorescent staining using antibodies against the corresponding epitope tags, as well as ALD to stain the parasite cytoplasm. (TIF) [file ppat.1010293.s002.tif]

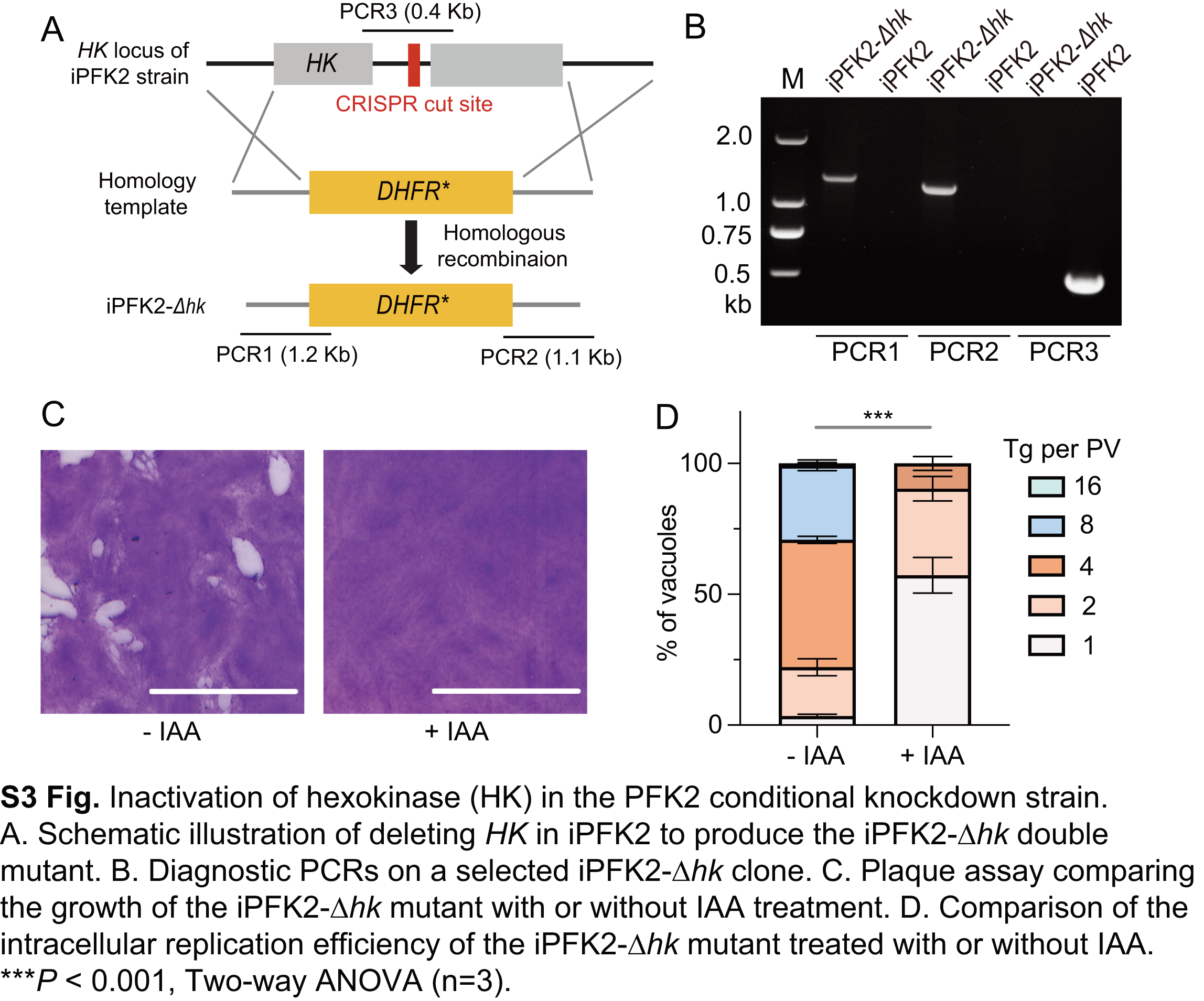

Supplement: S3 Fig — A. Schematic illustration of deleting HK in iPFK2 to produce the iPFK2-Δhk double mutant. B. Diagnostic PCRs on a selected iPFK2-Δhk clone. C. Plaque assay comparing the growth of the iPFK2-Δhk mutant with or without IAA treatment. D. Comparison of the intracellular replication efficiency of the iPFK2-Δhk mutant treated with or without IAA. ***P < 0.001, Two-way ANOVA (n = 3). (TIF) [file ppat.1010293.s003.tif]

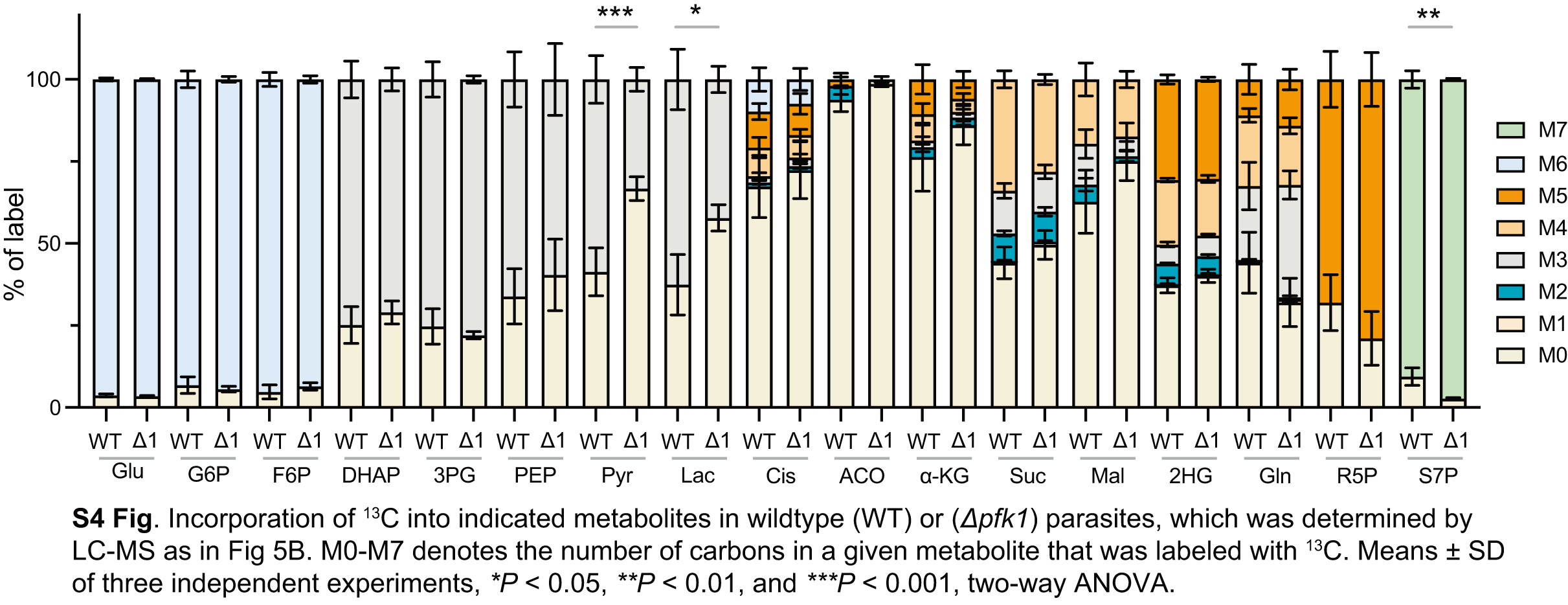

Supplement: S4 Fig — M0-M7 denotes the number of carbons in a given metabolite that was labeled with 13C. Means ± SD of three independent experiments, *P < 0.05, **P < 0.01, and ***P < 0.001, two-way ANOVA. (TIF) [file ppat.1010293.s004.tif]

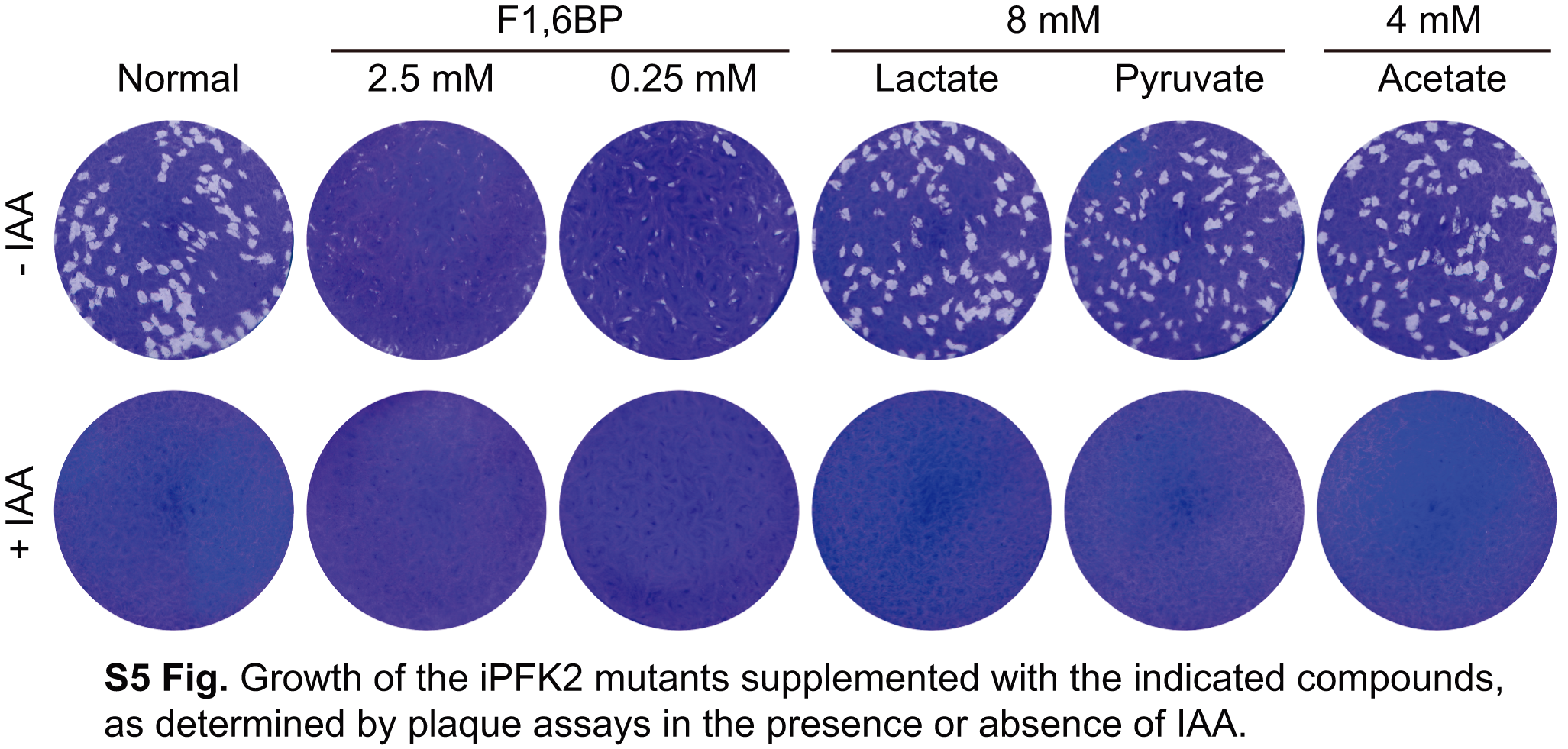

Supplement: S5 Fig — (TIF) [file ppat.1010293.s005.tif]

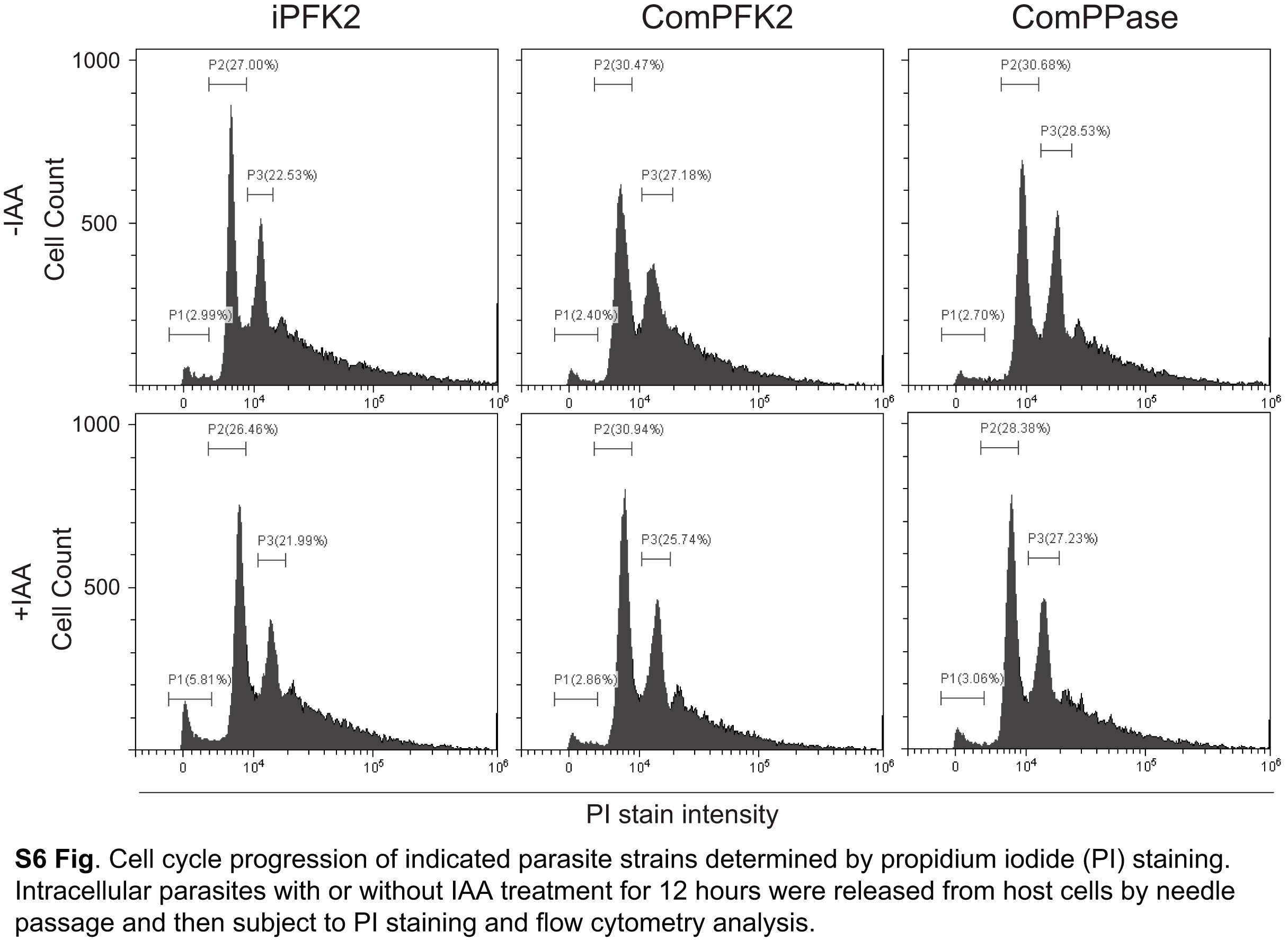

Supplement: S6 Fig — Intracellular parasites with or without IAA treatment for 12 hours were released from host cells by needle passage and then subject to PI staining and flow cytometry analysis. (TIF) [file ppat.1010293.s006.tif]

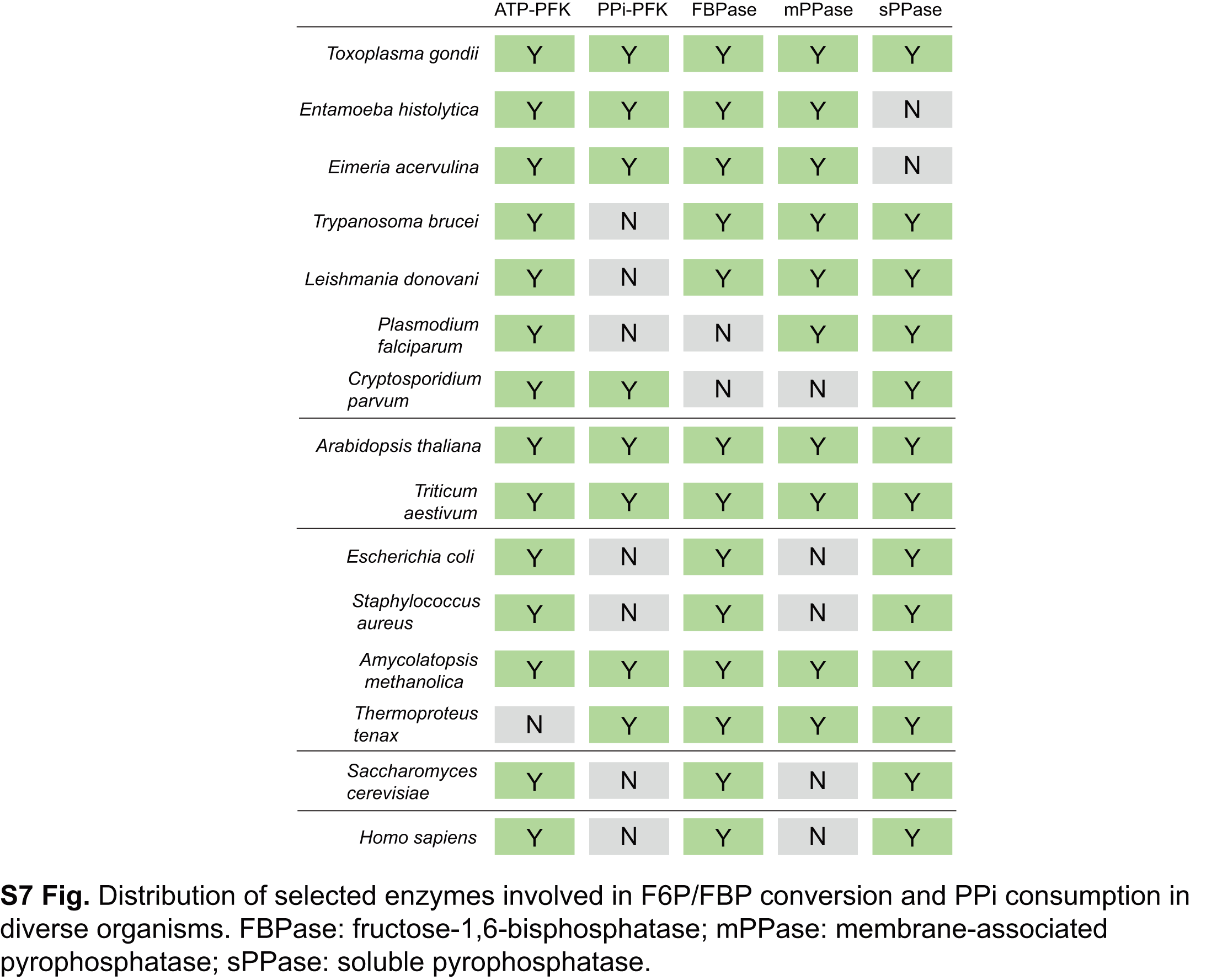

Supplement: S7 Fig — FBPase: fructose-1,6-bisphosphatase; mPPase: membrane-associated pyrophosphatase; sPPase: soluble pyrophosphatase. (TIF) [file ppat.1010293.s007.tif]
