# Supplementary material for: Essential role of pyrophosphate homeostasis mediated by the pyrophosphate-dependent phosphofructokinase in Toxoplasma gondii
Source: PLoS Pathog. 2022 Feb 1;18(2):e1010293. doi: 10.1371/journal.ppat.1010293 (PMC8836295; doi:10.1371/journal.ppat.1010293)
Supplement: S1 Table — (DOCX) [file ppat.1010293.s008.docx]

| **S1 Table. Plasmids used in this study** | | |
| --- | --- | --- |
| **Name of plasmids** | **Use** | **Construction methods** |
| pSAG1-Cas9-sgUPRT | Template for gene specific CRISPR plasmid construction | Reference 1 |
| pSAG1-Cas9-sgPFK1 | *PFK1* specific CRISPR plasmid for PFK1 knockout | Site-directed mutagenesis to replace the gRNA in pSAG1-Cas9-sgUPRT with gene specific gRNAs. |
| pSAG1-Cas9-sgPFK2-cKO | *PFK2* specific CRISPR plasmid for the iPFK2 construction |  |
| pSAG1-Cas9-sgHK | *HK* specific CRISPR plasmid |  |
| pAID::PFK2-HA | To construct the iPFK2 conditional knockdown strain | PCR derived fragments iPFK2-5H, AID-HXGPRT, iPFK2-3H were cloned into pUC19 |
| pTub::PFK2::Ty::*DHFR* | To construct the iPFK2 comp strain | The coding sequence of PFK2 was cloned into pPYK1::DHFR (Reference 2) |
| pTub::PFK1::Ty::*DHFR* | Express PFK1 in the iPFK2 strain | The coding sequence of PFK1 was cloned into pPYK1::DHFR |
| pTub::PFK2-N::Ty::*DHFR* | Express PFK2-N in the iPFK2 strain | The coding sequence of PFK2-N (1-595 aa) was cloned into pPYK1::DHFR |
| pTub::PFK2-C::Ty::*DHFR* | Express PFK2-C in the iPFK2 strain | The coding sequence of PFK2-C (597-1225 aa) was cloned into pPYK1::DHFR |
| pTub::PPase::Ty::*DHFR* | Express PPase in the iPFK2 strain | The coding sequence of PPase was cloned into pPYK1::DHFR |
| pPFK1::*DHFR* | Homologous template for *PFK1* replacement by *DHFR* | PCR derived fragments U5 PFK1, DHFR, U3 PFK1 were cloned into pUC19 |
| pHK::*DHFR* | Homologous template for *HK* replacement by *DHFR* | PCR derived fragments U5 HK, homology-DHFR, U3 HK were cloned into pUC19 |
| pEsumo-PFK1 | Prokaryotic expression of the full-length PFK1 protein | The coding sequence of PFK1 amplified from cDNA of the RH strain was cloned into the pE-SUMO vector |
| pEsumo-PFK2 | Prokaryotic expression of full-length PFK2 | The coding sequence of PFK2 amplified from cDNA of the RH strain was cloned into the pE-SUMO vector |
| pEsumo-PFK2N | Prokaryotic expression of PFK2-N | Deleting PFK2-C in pEsumo-PFK2 |
| pEsumo-PFK2C | Prokaryotic expression of PFK2-C | Deleting PFK2-N in pEsumo-PFK2 |
| pUC19 | Template for pUC19 amplification | From the Sibley Lab |

**References:**

1. Shen B, Brown KM, Lee TD, Sibley LD. Efficient gene disruption in diverse strains of Toxoplasma gondii using CRISPR/CAS9. mBio 5, e01114-01114 (2014).
2. Xia N, Ye S, Liang X, Chen P, Zhou Y, Fang R, Zhao J, Gupta N, Yang S, Yuan J, Shen B. Pyruvate Homeostasis as a Determinant of Parasite Growth and Metabolic Plasticity in Toxoplasma gondii. mBio. 10(3):e00898-19(2019).
