# Supplementary material for: Essential role of pyrophosphate homeostasis mediated by the pyrophosphate-dependent phosphofructokinase in Toxoplasma gondii
Source: PLoS Pathog. 2022 Feb 1;18(2):e1010293. doi: 10.1371/journal.ppat.1010293 (PMC8836295; doi:10.1371/journal.ppat.1010293)
Supplement: S2 Table — (DOCX) [file ppat.1010293.s009.docx]

| **S2 Table. Primers used in this study** | | |
| --- | --- | --- |
| **Primer** | **Sequence （5’ – 3’）** | **Use** |
| gRNA‐PFK1‐Fw | GCTCTTCAAAGTCTTCTGCGGTTTTAGAGCTAGAAATAGC | To construct the PFK1 specific CRISPR plasmid |
| gRNA‐PFK2‐Fw | GAGTTTGGAATTCTCTGTCGGTTTTAGAGCTAGAAATAGC | To construct the PFK2 specific CRISPR plasmid |
| gRNA‐HK‐Fw | GCAGTTATGGGCGCTCAATTGTTTTAGAGCTAGAAATAGC | To construct the HK specific CRISPR plasmid |
| gRNA‐UPRT‐Fw | GGCGTCTCGATTGTGAGAGCGTTTTAGAGCTAGAAATAGC | To construct the UPRT specific CRISPR plasmid |
| gRNA-R | AACTTGACATCCCCATTTAC | To construct gene specific CRISPR plasmids |
| DHFR-F | cttcagaaccCAGGCTGTAAATCCCGTGAGTC | Amplification of DHFR for p PFK1::DHFR construction |
| DHFR-R | cgtgttcattcGATTCCGTCAGCGGTCTGTC |  |
| U5 PFK1-F | attgtactgagagtgcaccaCTAGTGGCACAACAGCTGTGTGC | Amplification of 5’-homology of PFK1 for p PFK1::DHFR construction |
| U5 PFK1-R | ttacagcctgGGTTCTGAAGCGTGACGAGAA |  |
| U3 PFK1-F | gacggaatcGAATGAACACGAAGCAGAGAATCTT | Amplification of 3’-homology of PFK1 for p PFK1::DHFR construction |
| U3 PFK1-R | cgactctagaggatccccggCTGTTAAAGCGAAAAGTGAAAGGTT |  |
| PFK1-PCR1-F | CTGACATTCTCTGGCAAAAG | PCR1 of RHΔHX-Δpfk1 |
| PFK1-PCR1-R | CCAGTCATGGACGAGATCG | PCR1 of RHΔHX-Δpfk1 and iPFK2∆hk |
| PFK1-DHFR-3H-F | GACTCTTCATGTGGCATTTC | PCR2 of RHΔHX-Δpfk1 and iPFK2∆hk |
| PFK1-DHFR-3H-R | GTGAAACTCTGTGCGGCCGC | PCR2 of RHΔHX-Δpfk1 |
| PFK1-PCR3-F | CAGCATCCGTCGCTGTGGAC | PCR3 of RHΔHX-Δpfk1 |
| PFK1-PCR3-R | CCGACGCGGTCTCCGGTGGC |  |
| pUC19 Forward | CCGGGGATCCTCTAGAGTCG | Amplification of pUC19 for pPFK1::DHFR, pHK::DHFR, pAID:: PFK2-HA and pHK::DHFR construction |
| pUC19 Reverse | TGGTGCACTCTCAGTACAATCTGC |  |
| iPFK2-5H-F | tactgagagtgcaccatatgGAAGAGCTTGTCTAGCCTTCATAGTATT | Amplification of 5’-homology of PFK2 for pAID:: PFK2-HA construction |
| iPFK2-5H-R | accatcctaggTTGGTCTCTGCATGCAGCC |  |
| AID-HXGPRT-F | agagaccaaCCTAGGATGGTGAGCGCTAGC | Amplification of AID-HXGPRT for pAID:: PFK2-HA construction |
| AID-HXGPRT-R | ctctgCCCATTCGCCATTCAGGC |  |
| iPFK2-3H-F | tgaatggcgaatgggCAGAGGCTCTGGGACAGACG | Amplification of 3’-homology of PFK2 for pAID:: PFK2-HA construction |
| iPFK2-3H-R | ctagaggatccccgggtaccCGTCACGTCAGGAGAATGATGA |  |
| iPFK2-PCR1-F | CTCTCTGTGTCTGTGTGACTC | PCR1 of iPFK2 |
| iPFK2-PCR1-R | GGCAAGAGACCATCACGTTC | PCR1 of iPFK2 and iPPase |
| iPFK2-PCR2-F | GCGTTGGCCTACGTGACTTG | PCR2 of iPFK2 and iPPase |
| iPFK2-PCR2-R | CTGAAAGATGGGGAAGCGCAAC | PCR2 of iPFK2 |
| iPFK2-PCR3-F | GAGCGACCGGTGCTGAGTTG | PCR3 of iPFK2 |
| iPFK2-PCR3-R | CGGCGTGAGGTGAACAAACAC |  |
| HK homology-DHFR-F | ccttctcaagttcaaCAGGCTGTAAATCCCGTGAGTC | Amplification of DHFR of HK for pHK::DHFR construction |
| HK homology-DHFR-R | aacacGATTCCGTCAGCGGTCTGTC |  |
| U5 HK-F | attgtactgagagtgcaccaCGCGAAAAAAACAGTGAGGC | Amplification of 5’-homology of HK for pHK::DHFR construction |
| U5 HK-R | gcctgTTGAACTTGAGAAGGAGGATCTACG |  |
| U3 HK-F | accgctgacggaatcGTGTTGGGGCCAGGATGC | Amplification of 3’-homology of HK for pHK::DHFR construction |
| U3 HK-R | cgactctagaggatccccggGACAACACACAGAACAAAACAGGC |  |
| HK-PCR1-F | CCTCTTGCGGCTCTACTTCG | PCR1 of iPFK2∆hk |
| HK-PCR2-R | CGTCCCGGTCCTCTCTGTC | PCR2 of iPFK2∆hk |
| HK-PCR3-F | CGGTAACTTTCCCGAGAGG | PCR3 of iPFK2∆hk |
| HK-PCR3-R | CCAACCTGACAGAGGCAAGC |  |
| PFK2-CDS-F | gatctaaaatggtgagcaagATGACTTTCTTGTCCTTCTTTAAGTGC | Amplification of PFK2 CDS for pTub::PFK2::DHFR construction |
| PFK2-CDS-R | tcctggttcgtgtggacctcTTGGTCTCTGCATGCAGCC |  |
| PFK1-CDS-F | gatctaaaatggtgagcaagATGGCGTATCCTCCAAGTGGC | Amplification of PFK1 CDS for pTub::PFK1::DHFR construction |
| PFK1-CDS-R | tcctggttcgtgtggacctcGAAGTCTTCGCGTTTCGAGAAT |  |
| (1-595aa)N-PFK2-F | gatctaaaatggtgagcaagATGACTTTCTTGTCCTTCTTTAAGTGC | Amplification of N-terminal truncated PFK2 CDS for pTub::PFK2-N::DHFR construction |
| (1-595aa)N-PFK2-R | tcctggttcgtgtggacctcCGACGCCGTAGACGGCGC |  |
| (597-1225aa)C-PFK2-F | gatctaaaatggtgagcaagATGCTGTTGTCGAGTTCCTCTCC | Amplification of C-terminal truncated PFK2 CDS for pTub::PFK2-C::DHFR construction |
| (597-1225aa)C-PFK2-R | tcctggttcgtgtggacctcTTGGTCTCTGCATGCAGCC |  |
| PPase-CDS-F | gatctaaaatggtgagcaagATGCAGTCTGCACCTCTGGC | Amplification of PPase CDS for pTub::PPase::DHFR construction |
| PPase-CDS-R | tcctggttcgtgtggacctcCGGTAGCCACAACTTTTGCTTC |  |
| p-Tub-DHFR-F | GAGGTCCACACGAACCAGGACCCGCTCGATTAATAAATGCAGCCCACAGAAGC | Amplification of vector for p pTub::PFK2::DHFR construction |
| p-Tub-DHFR-R | CTTGCTCACCATTTTAGATCTAAAAGG |  |
| Comp-pfk2-PCR4-F | CGTCTTATCCGTCGCTGTAC | PCR4 of ComPFK2 |
| Comp-PPase-PCR4-F | GGCGCTCTGGCGATGATCG | PCR4 of ComPPase |
| Comp-pfk1-PCR4-F | GAAGGACATTCGACAGCAGC | PCR4 of ComPFK1 |
| Comp-pfk2N-PCR4-F | GATTCACACTGAGAAGCTG | PCR4 of ComPFK2-N |
| Comp-pfk2C-PCR4-F | CTCGCTCCGGGCGTCTTATCC | PCR4 of ComPFK2-C |
| Comp-PCR4-R | CGAGCGGGTCCTGGTTCGTG | PCR4 of ComPFK2, iPFK2/PFK1, iPFK2/N-PFK2，iPFK2/C-PFK2, iPFK2/PPase |
| PCR5-5'-in-upUPRT-F | GGGTGCCTACGTTCTTCTAC | PCR5 of ComPFK2, iPFK2/PFK1，iPFK2/N-PFK2，iPFK2/C-PFK2, iPFK2/PPase |
| PCR5-3'-in-downUPRT-R | GTCCCCAGGTAGCGAGAACG |  |
| pE sumo-vector-F | TAAACTAGAGGATCCGAATTCG | Amplification of the vector for pEsumo-PFK1/2 construction |
| pE sumo-vector-R | ACCTCCAATCTGTTCGCGGTG |  |
| F-His-PFK1 | CGAACAGATTGGAGGTATGGCGTATCCTCCAAGTG | Amplification of the PFK1 CDS for pEsumo-PFK1 construction |
| R-His-PFK1 | CGGATCCTCTAGTTTAGAAGTCTTCGCGTTTCGAGAATCG |  |
| F-His-PFK2 | CCGCGAACAGATTGGAGGTATGACTTTCTTGTCCTTCTTT | Amplification of the PFK2 CDS for pEsumo-PFK2 construction |
| R-His-PFK2 | AATTCGGATCCTCTAGTTTATTGGTCTCTGCATGCAGCC |  |
| F-C-PFK2 | CGCGAACAGATTGGAGGTTTGTCGAGTTCCTCTCCCCAG | For the construction of pEsumo-PFK2C |
| R-C-PFK2 | ACCTCCAATCTGTTCGCGGTG |  |
| F-N-PFK2 | GCGCCGTCTACGGCGTCGTAAACTAGAGGATCCGAATTC | For the construction of pEsumo-PFK2N |
| R-N-PFK2 | CGACGCCGTAGACGGCGC |  |
| GAPDH1-F | TGAAGGGAATCATCAGCTACAC | qPCR for parasite load determination |
| GAPDH1-R | TGTCAAACACGGAGGAGAAC |  |
